# AREG^+^ regulatory T cells mediating myocardial repair and neovascularization after myocardial infarction

**DOI:** 10.1186/s10020-025-01281-8

**Published:** 2025-06-11

**Authors:** Yan Wang, Jiao Li, Yu Zhang, Pingping He, Weiwei Liu, Weirong Zeng, Chaofu Li, Yixuan Gao, Yongchao Zhao, Changyin Shen, Wenming Chen, Yunhang Li, Ranzun Zhao, Bei Shi

**Affiliations:** 1 Department of Cardiology, Affiliated Hospital of Zunyi Medical University, Zunyi, 563000 China; 2 Department of Cardiology, Guizhou Provincial Staff Hospital, Guiyang, 550000 China

**Keywords:** Acute myocardial infarction, Neovascularization, AREG, Regulatory T cells, FoxM1

## Abstract

Enhancing tissue repair and angiogenesis post-Acute myocardial infarction (AMI) is critical to improving cardiac function and preventing heart failure. Recent studies have highlighted the interaction between immune cells and endothelial cells in post-AMI angiogenesis, yet the specific contributions of different immune cell subsets remain poorly understood. Emerging evidence suggests the presence of a subset of regulatory T cells (Tregs) expressing Amphiregulin (AREG) in infarcted myocardial tissue, with AREG acting as a paracrine protein that plays a key role in angiogenesis. However, whether AREG⁺ Tregs mediate post-AMI angiogenesis and the underlying molecular mechanisms remain unclear. In this study, we demonstrate that Treg activation promotes tissue repair and improves cardiac function after AMI, although it does not significantly impact angiogenesis or endothelial cell behaviors. Notably, overexpression of AREG⁺ Tregs stimulates robust neovascularization post-AMI, reduces myocardial fibrosis, and further enhances cardiac function. Our in vitro data reveal that AREG⁺ Tregs secrete not only AREG but also angiogenic factors such as VEGF and FGF, which promote endothelial cell proliferation, migration, and tube formation. RNA sequencing analysis identifies FoxM1 as a key regulator of this angiogenic process in cardiac microvascular endothelial cells (CMECs). Importantly, inhibition of AREG in Tregs abolishes the FoxM1-dependent angiogenic response.In summary, we identified AREG⁺ Tregs as a distinct cell population driving neovascularization post-AMI, with FoxM1-mediated angiogenesis in CMECs. This may provide a foundation for harnessing specific Treg subsets for cardioprotection following AMI.

**Supplementary Information:**

The online version contains supplementary material available at 10.1186/s10020-025-01281-8.

## Introduction

Acute myocardial infarction (AMI) triggers a complex immune-inflammatory response, with immune cells accumulating in the infarcted area to promote tissue angiogenesis by secreting proteins that signal nearby endothelial cells (ECs) expressing cognate receptors (Lazzerini et al. 2019). Despite this, the full complexity of the functional adaptive responses to cellular crosstalk and neovascularization following AMI remains incompletely understood. Among the immune cells activated after AMI, regulatory T cells (Tregs), defined as CD4^+^ CD25^+^ FoxP3^+^ T lymphocytes, play a critical role in cardiovascular health, particularly in atherosclerosis and abdominal aortic aneurysms (Longevity 2024; Blanco-Dominguez et al. 2022). Post-AMI, Tregs are recruited to the heart, where they contribute to myocardial repair and maintain structural stability by promoting fibrotic repair (Zhuang et al. 2022). Evidence suggests that Tregs support myocardial repair primarily by regulating monocyte and macrophage differentiation, with Treg depletion exacerbating myocardial injury (Li et al. 2022; Wang et al. 2022; Zhu et al. 2022). Clinically, patients with coronary artery disease and low circulating Tregs have a higher risk of perioperative cardiovascular events following percutaneous coronary intervention (Johnson et al. 2019).

However, the role of Tregs in angiogenesis remains controversial, varying across tissues and microenvironments. While some studies suggest that Tregs promote angiogenesis, such as in a lower extremity ischemia model where they secrete IL-10 to facilitate revascularization (Liu and Wei 2021; Berasain and Avila 2014), others report detrimental effects of Tregs on revascularization, particularly in heart failure models (Zaiss et al. 2015; Sugita et al. 2021). These discrepancies may stem from variations in Treg phenotypes or experimental models. Therefore, whether Tregs contribute to neovascularization after AMI and the underlying mechanisms remain unclear.

Recently, mRNA sequencing of Tregs from heart, spleen, and liver tissues after AMI revealed significantly higher Amphiregulin (AREG) expression in heart-derived Tregs (Zhuang et al. 2022). AREG, a member of the EGF family, acts on the Epidermal Growth Factor Receptor (EGFR) to regulate cell proliferation, apoptosis, and migration (Florentin et al. 2022). Notably, Tregs have been shown to promote cardiomyocyte proliferation and myocardial repair in a paracrine manner, with AREG playing a central role (Astarita et al. 2023). Given this, we hypothesize that AREG-expressing Tregs may also regulate post-AMI neovascularization. we found that AREG was highly expressed in the Tregs. In both functional studies of cells and animal experiments, AREG Tregs was found to promote angiogenesis. In addition, AREG promotes neovascularization post-AMI by regulating Forkhead Box Protein M1 (FoxM1).

## Methods

The data, analytical methods, and study materials are available from the corresponding author upon reasonable request.

Detailed Materials and Methods are provided in the online Supplemental Material.

### Mice

C57 BL/6 J mice were obtained from the experimental animal center of Zunyi Medical University and were housed and bred under specific pathogen-free conditions. All surgeries were performed on 8 to 10-week-old male mice.

### Additional methods

The expanded Methods section in the online Supplemental Material includes information on the treatment and group, the description of flow cytometric analyses, quantification of absolute cell numbers, quantitative real-time polymerase chain reaction (PCR), echocardiographic analyses of cardiac function, infarct size assessment, western blotting, cell culture, and enzyme-linked immunosorbent assay.

### Statistical analysis

Statistical analysis was mainly performed using SPSS 21.0 statistical software package (IBM, Armonk, NY, USA) and GraphPad Prism 7.0 (GraphPad Software Inc., San Diego, CA, USA). The data were normally distributed and are expressed as the mean ± SD. Comparisons between two groups were made using the Mann–Whitney U test, whereas one-way analysis of variance was used to compare multiple groups. Statistical significance was set as *P* < 0.05.

## Results

### Temporal dynamics of AREG^+^ Tregs infiltration in the myocardium after AMI

**Fig. 1:**
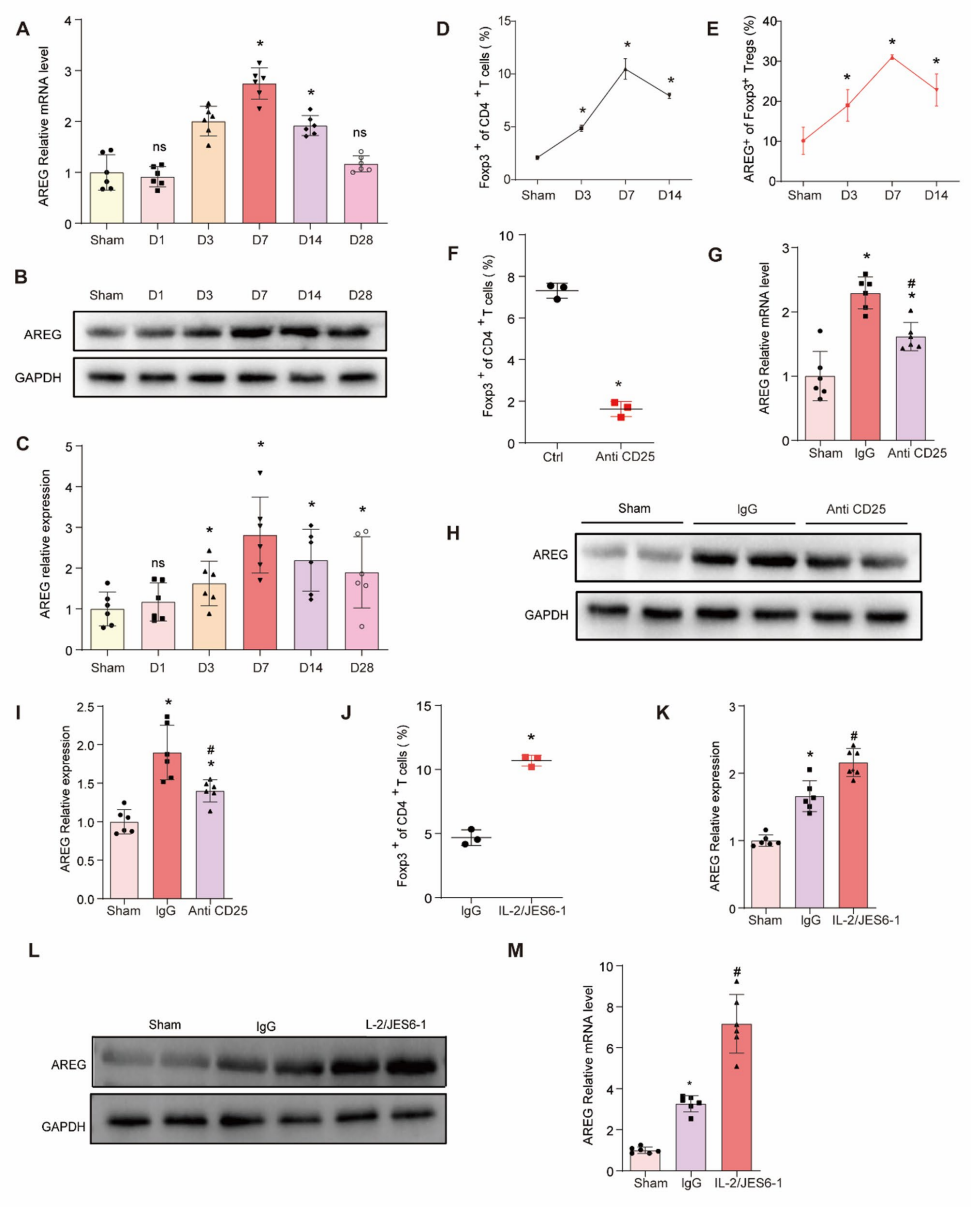
Changes in AREG expression after myocardial infarction. **A **The relative mRNA level of AREG at day 1,day 3, day 5, day 7 and day 14 after AMI or Sham operation. *n* = 6 per group. ^*^P < 0.05 vs. Sham. **B **AREG protein levels in the infarcted myocardium were quantified by Western blotting at Sham, day 1,day 3, day 5, day 7 and day 14 after AMI. **C **Summary data of B. *n* = 6 per group. ^*^P < 0.05 vs. Sham. **D ** The proportions of Tregs in the hearts at day 1, day 3, day 7 and day 14 after AMI or Sham operation. *n* = 6 per group. ^*^P < 0.05 vs. Sham. **E **The proportions of AREG^+^ Tregs in the hearts at day 1, day 3, day 7 and day 14 after AMI or Sham operation. *n* = 6 per group. ^*^P < 0.05 vs. Sham. **F **The proportions of Tregs in the hearts in Ctrl and Anti CD25 group. *n* = 6 per group. ^*^P < 0.05 vs. Ctrl. **G **Relative mRNA level of AREG in Sham, lgG and Anti CD25 group. *n* = 6 per group. ^*^P < 0.05 vs. Sham, ^#^*P* < 0.05 vs. lgG. **H **AREG protein levels in the infarcted myocardium were quantified by Western blotting in Sham, lgG and Anti CD25 group respectively. **I **Summary data of H. *n* = 6 per group. ^*^P < 0.05 vs. Sham, ^#^*P* < 0.05 vs. lgG. **J **The proportions of Tregs in the hearts lgG and IL-2/JES6-1. *n* = 6 per group. ^*^P < 0.05 vs. lgG. **K **Relative mRNA level of AREG in Sham, lgG and IL-2/JES6-1 group. *n* = 6 per group. ^*^P < 0.05 vs. Sham, ^#^*P* < 0.05 vs. lgG. **L **AREG protein levels in the infarcted myocardium were quantified by Western blotting in Sham, lgG and IL-2/JES6-1 group respectively. **M **Summary data of H. *n* = 6 per group. ^*^P < 0.05 vs. Sham, ^#^*P* < 0.05 vs. lgG

Previous studies have shown that AREG-positive Tregs preferentially accumulate in the heart following myocardial infarction (MI) compared to other tissues, such as the liver (Zhuang et al. 2022). However, the temporal dynamics of AREG expression and the infiltration of AREG⁺ Tregs during post-infarction recovery remain unclear. To investigate this, we examined the mRNA and protein levels of AREG in the infarcted region of the heart at various time points (1, 3, 7, 14, and 28 days) after MI, as well as in the Sham group. The results revealed that AREG expression gradually increased, peaking on day 7 post-MI, before decreasing but remaining higher than in the Sham group (Fig. 1A-C). Flow cytometry showed a significant increase in Treg numbers on day 7 post-MI compared to the Sham group (Fig. 1D), with AREG⁺ Tregs also peaking on day 7 (Fig. 1E).

To further clarify the origin of AREG in infarcted cardiac tissues, we observed the effects of partial Treg depletion using anti-CD25 antibody in vivo. The results showed that after AMI, the anti CD25 antibody partially cleared Treg in infarcted heart tissues of mice compared to the Ctrl group (Fig. 1F). Interestingly, In a mouse AMI model, AREG expression was significantly reduced in the infarcted myocardium after Treg depletion, but still higher than in the Sham group (Fig. 1G-I). Conversely, intraperitoneal injection with IL-2/JES6-1 complex effectively stimulated Tregs expansion in the infarcted area of mouse myocardium (Fig. 1J). Additionally, IL-2/JES6-1 complex-mediated Treg expansion significantly increased AREG expression in the infarcted myocardium compared to the IgG group (Fig. 1K-M). These findings suggest that AREG in infarcted cardiac tissues is at least partially derived from infiltrating Tregs.

### Tregs improve myocardial infarction cardiac function, but have no significant effect on post-AMI neovascularization

**Fig. 2:**
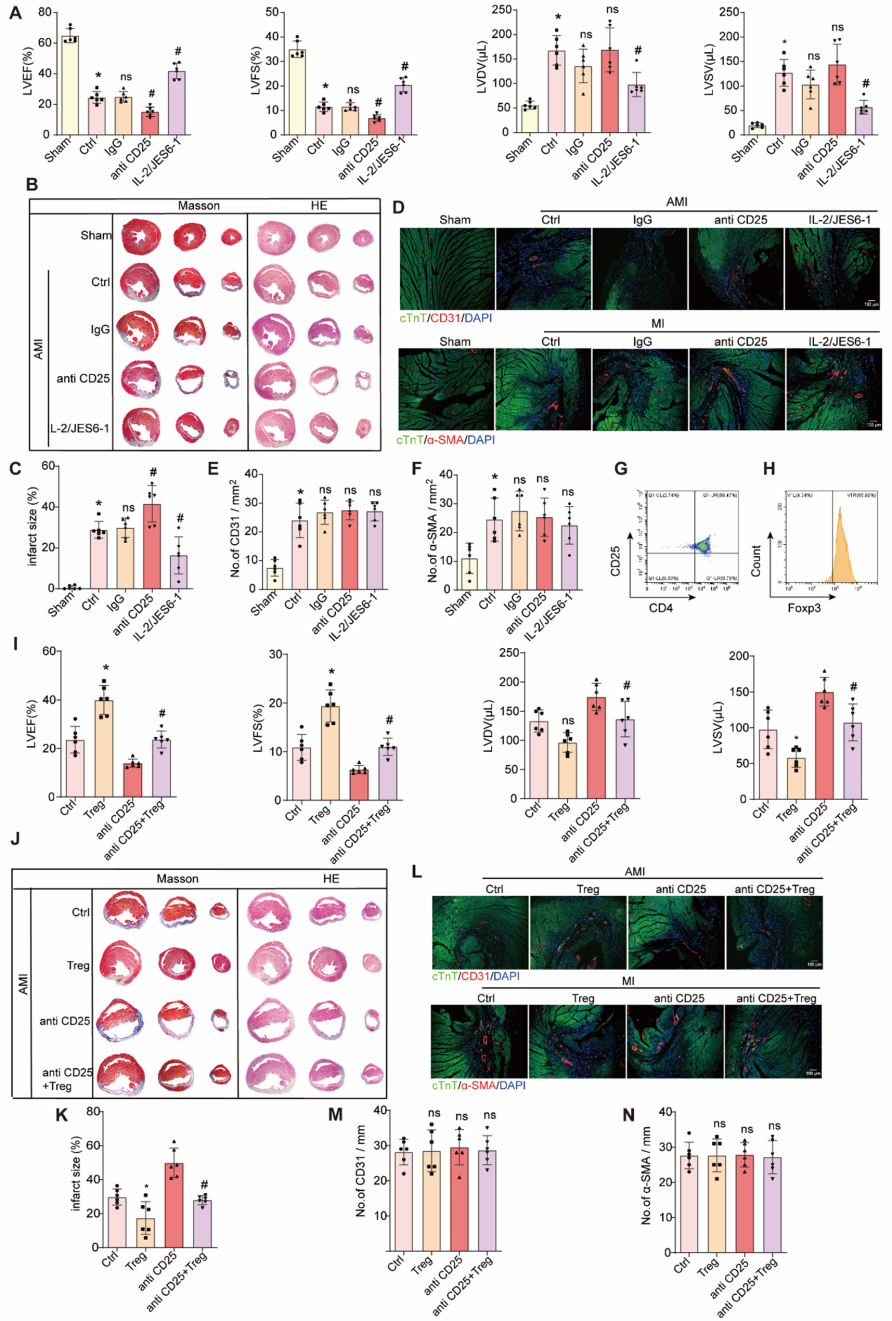
Effect of Tregs on cardiac function and neovascularization after AMI. **B ** LVEF, LVFS, LVDV, and LVSV at day 28 post-AMI by echocardiography in sham, Ctrl, lgG, anti CD25 and IL2/JES6 group. *n* = 6 per group. ^*^P < 0.05 vs. Sham, ^#^*P* < 0.05 vs. Ctrl. **B **Representative Masson’s trichrome and HE staining of day 28 post-AMI heart sections from 6 independent experiments. Scale bars, 2 mm. **C **Quantification of infarct size on day 28 post- AMI hearts. *n* = 6 per group. ^*^P < 0.05 vs. Sham, ^#^*P* < 0.05 vs. Ctrl. **D **Immunostaining with an anti-CD31 or anti-αSMA antibody on day 28 post-AMI heart sections. Scale bars, 50 mm. **E **Quantification of CD31 on day 28 post-AMI hearts. *n* = 6 per group. ^*^P < 0.05 vs. Sham, ^#^*P* < 0.05 vs. Ctrl. **F ** Quantification of α-SMA on day 28 post-AMI hearts. *n* = 6 per group. ^*^P < 0.05 vs. Sham, ^#^*P* < 0.05 vs. Ctrl. **G **and** H **Representative flow cytometry pseudo color plot and percentages of FoxP3^+^ gated in CD45^+^CD25^+^CD4^+^ cells (Tregs). **I **LVEF, LVFS, LVDV, and LVSV at day 28 post-AMI by echocardiography in Ctrl, Treg, anti CD25 and anti CD25 + Treg group. *n* = 6 per group. ^*^P < 0.05 vs. Ctrl, ^#^*P* < 0.05 vs. Treg. **J **Representative Masson’s trichrome and HE staining of day 28 post-AMI heart sections from 6 independent experiments. Scale bars, 2 mm. **K **Quantification of infarct size on day 28 post-AMI hearts. *n* = 6 per group. ^*^P < 0.05 vs. Ctrl, ^#^*P* < 0.05 vs. Treg. **L **Immunostaining with an anti-CD31 or anti-αSMA antibody on day 28 post-AMI heart sections. Scale bars, 50 mm **M **Quantification of CD31 on day 28 post-AMI hearts. *n* = 6 per group. ^*^P < 0.05 vs. Ctrl, ^#^*P* < 0.05 vs. Treg. **N **Quantification of α-SMA on day 28 post-AMI hearts. *n* = 6 per group. ^*^P < 0.05 vs. Ctrl, ^#^*P* < 0.05 vs. Treg

Tregs have been shown to improve cardiac function and ventricular remodeling after AMI (Zhuang et al. 2022; Alshoubaki et al. 2024). To assess their role in cardiac function, myocardial infarction area, and neovascularization, we examined the effects of Treg depletion and expansion in vivo. Cardiac ultrasound results (Fig. 2A) indicated significantly lower LVEF and LVFS, and higher LVDV and LVSV in the Ctrl group compared to the Sham group (*P* < 0.05). In the anti-CD25 group, LVEF and LVFS were further reduced, while LVDV and LVSV were significantly increased (*P* < 0.05). In contrast, Treg expansion in the IL-2/JES6-1 group significantly improved cardiac function, with higher LVEF and LVFS, and lower LVDV and LVSV (*P* < 0.05).Histological analysis (Fig. 2B and C) revealed a significantly larger myocardial infarct area in the Ctrl and IgG groups compared to the Sham group (*P* < 0.05), with a further increase in the anti-CD25 group. In the IL-2/JES6-1 group, the infarct area was significantly reduced (*P* < 0.05). These results suggest that partial Treg depletion worsened cardiac function and increased myocardial infarct size, whereas Treg expansion improved both. Neovascularization was assessed by immunofluorescence staining (Fig. 2D-F), showing an increase in CD31- and α-SMA-positive blood vessels in the infarct junction of the Ctrl and IgG groups compared to the Sham group (*P* < 0.05). However, no significant differences were observed in the number of blood vessels in the infarct area between the anti-CD25 group and the IL-2/JES6-1 group (*P* > 0.05), indicating that Treg modulation did not affect angiogenesis in this model.

To further explore the potential of exogenous Treg transplantation, we isolated and cultured Tregs and transferred them into AMI mice. Flow cytometry confirmed high purity of the isolated Tregs (Fig. 2G and H). Post-transplantation, cardiac ultrasound showed that LVEF and LVFS were significantly higher (*P* < 0.05), and LVSV was significantly lower (*P* < 0.05) in the Treg group compared to the Ctrl group. Anti-CD25 further deteriorated cardiac function, but Treg transplantation reversed this effect (Fig. 2I). Histological analysis (Fig. 2J and K) showed that Treg transfer significantly reduced myocardial fibrosis compared to the Ctrl and anti-CD25 groups (*P* < 0.05). However, immunofluorescence staining (Fig. 2L-N) revealed no significant differences in the number of CD31-positive capillaries or α-SMA-positive small arteriolar vessels between the Treg and Ctrl groups, as well as between the anti-CD25 and anti-CD25 + Tregs groups (*P* > 0.05).These results indicate that while Treg transfer significantly reduces infarct size and improves cardiac function after AMI, it does not have a significant effect on post-infarction neovascularization, similar to the effects of endogenous Treg amplification.

### AREG⁺ Tregs promote post-infarction neovascularization and improve cardiac function

Tregs have been shown to play a reparative role in myocardial healing by modulating macrophage activity after AMI (Weirather et al. 2014). Our data also confirmed that Tregs ameliorate myocardial fibrosis and improve cardiac function post-AMI, but have no significant effect on neovascularization. Given the functional heterogeneity of Tregs in different microenvironments, we hypothesized that Tregs expressing specific receptors might have enhanced myocardial repair effects and mediate neovascularization.

Previous studies identified AREG⁺ Tregs in cardiac tissue post-AMI through single-cell sequencing (Zhuang et al. 2022). In our study, the upregulation of AREG expression peaked at 7 days post-AMI, coinciding with the peak of Treg infiltration, and AREG⁺ Tregs were significantly increased at this time point. Interestingly, Tregs deficient in GRP174 were shown to be essential for neovascularization in ischemic injury by upregulating AREG expression (Liu et al. 2022). However, the exact mechanism by which AREG contributes to myocardial repair and neovascularization remains unclear.

**Fig. 3:**
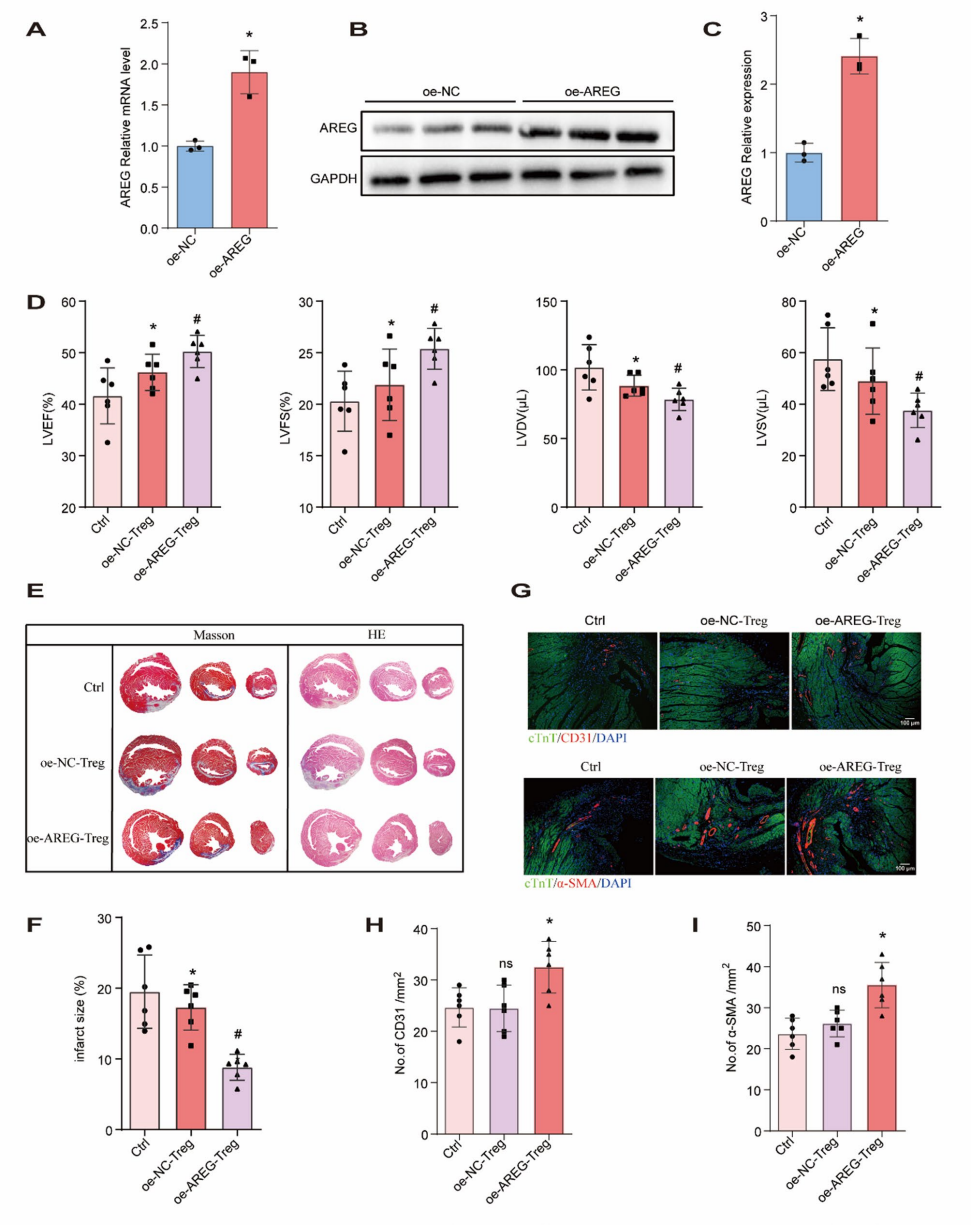
Effect of AREG^+^ Tregs on cardiac function and neovascularization after AMI. **A** The relative mRNA level of AREG in oe-NC and oe-AREG group. *n* = 6 per group. ^*^P < 0.05 vs. oe-NC. **B **AREG protein levels in the infarcted myocardium were quantified by Western blotting at oe-NC and oe-AREG group. **C **Summary data of B. *n* = 6 per group. ^*^P < 0.05 vs. oe-NC. **D **LVEF, LVFS, LVEF, LVFS, LVDV, and LVSV at day 28 post-AMI by echocardiography in Ctrl, oe-NC-Treg, oe-AREG-Treg group. *n* = 6 per group. ^*^P < 0.05 vs. Ctrl, ^#^*P* < 0.05 vs. oe-NC-Treg. **E **Representative Masson’s trichrome and HE staining of day 28 post-AMI heart sections from 6 independent experiments. Scale bars, 2 mm. **F **Quantification of infarct size on day 28 post-AMI hearts. *n* = 6 per group. *n* = 6 per group. ^*^P < 0.05 vs. Ctrl, ^#^*P* < 0.05 vs. oe-NC-Treg. **G **Immunostaining with an anti-CD31 or anti-αSMA antibody on day 28 post-AMI heart sections. cale bars, 50 mm. **H **Quantification of CD31 on day 28 post-AMI hearts. *n* = 6 per group. ^*^P < 0.05 vs. Ctrl, ^#^*P* < 0.05 vs. oe-NC-Treg. **I **Quantification of α-SMA on day 28 post-AMI hearts.*n* = 6 per group,^*^P < 0.05 vs. Ctrl, ^#^*P* < 0.05 vs. oe-NC-Treg

To explore the role of AREG⁺ Tregs in cardiac function and neovascularization, we performed gain-of-function experiments in mice. Splenic Tregs were isolated and transfected with lentiviruses carrying AREG or its negative control (NC), and successful transfection was confirmed by RT-PCR and Western blot (Fig. 3A and C).Tregs overexpressing AREG (oe-AREG-Tregs) were transferred into AMI mice by tail vein injection (1 × 10^6 cells), and their effects on myocardial infarction and neovascularization were assessed. Compared to the Ctrl group and the oe-NC-Treg group, the oe-AREG-Treg group showed significantly improved cardiac function (Fig. 3D), reduced infarct area (Fig. 3E F), and enhanced neovascularization in the infarct border zone (Fig. 3G and I).These results suggest that AREG⁺ Tregs promote cardiac repair and neovascularization after AMI. In the next step, we further investigated whether oe-AREG-Tregs could induce neovascularization in vitro.

### AREG⁺ Tregs promote the proliferation, migration, and tube formation of CMECs

**Fig. 4:**
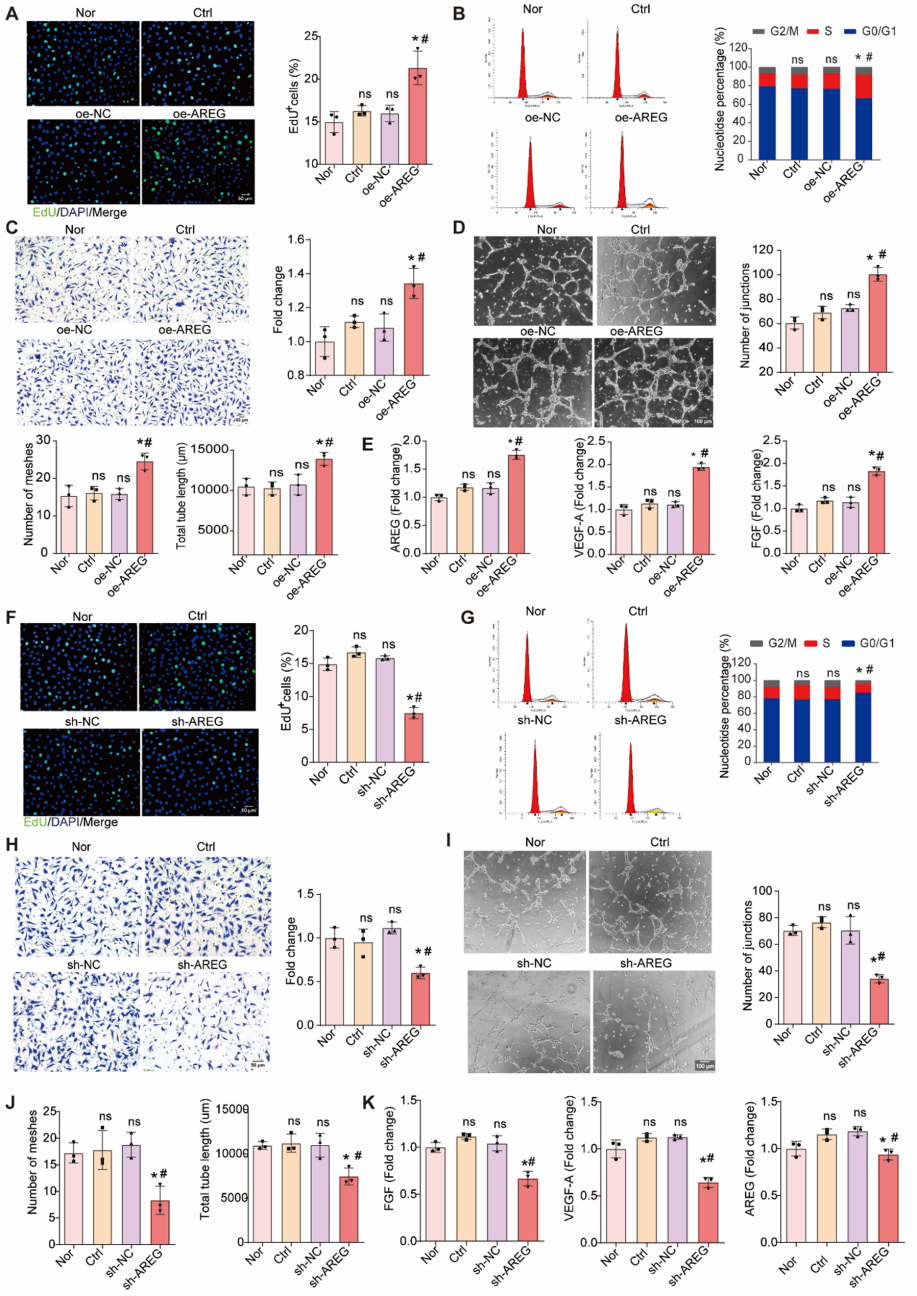
AREG is an important factor in the promotion of CMECs proliferation, migration and tube formation by Tregs. **A **Representative EdU staining images of CMECs, quantification of EdU-positive cells is shown on the right. *n* = 3 per group. ^*^P< 0.05*vs* Nor,^#^*P*< 0.05 *vs* Ctrl. **B **Flow cytometry analysis of the cell cycle in CEMCs in the Nor, Ctrl, oe-NC, oe-AREG groups. The proportion of cells in G0/G1, S, and G2/M phases is displayed in the accompanying bar graph. *n* = 3 per group. ^*^P< 0.05 *vs* Nor,^#^*P*< 0.05 *vs* Ctrl. **C **Representative migration assay results (crystal violet staining) with corresponding quantification of fold change in migrated cells.^*^P< 0.05 *vs* Nor,^#^*P*< 0.05 *vs* Ctrl. **D **and** E **Tube formation assay illustrating the effects of AREG overexpression on angiogenesis, with quantification of the number of junctions, meshs and total tuble length.^*^P< 0.05 *vs* Nor,^#^*P*< 0.05 *vs* Ctrl. **F **Expression of AREG, VEGF-A, and FGF in CMECs co-cultured with Tregs under the specified conditions.^*^P< 0.05 *vs* Nor,^#^*P*< 0.05 *vs* Ctrl. **G **Representative EdU staining images of CMECs in the Nor, Ctrl, sh-NC, sh-AREG groups, and quantification of EdU-positive cells is shown on the right. *n* = 3 per group. ^*^P< 0.05 *vs* Nor,^#^*P*< 0.05 *vs* Ctrl. **H **Flow cytometry analysis of the cell cycle in CEMCs in the Nor, Ctrl, sh-NC, sh-AREG groups. The proportion of cells in G0/G1, S, and G2/M phases is displayed in the accompanying bar graph. *n* = 3 per group. ^*^P< 0.05 *vs* Nor,^#^*P*< 0.05 *vs* Ctrl. **I **Representative migration assay results (crystal violet staining) with corresponding quantification of fold change in migrated cells.^*^P< 0.05 *vs* Nor,^#^*P*< 0.05 *vs* Ctrl. **J **and **K **Tube formation assay illustrating the effects of CMECs in the Nor, Ctrl, sh-NC, sh-AREG groups, with quantification of the number of junctions, meshs and total tuble length.^*^P< 0.05 *vs* Nor,^#^*P*< 0.05 *vs* Ctrl. **L **Expression of AREG, VEGF-A, and FGF in CMECs co-cultured with Tregs under the specified conditions.^*^P< 0.05 *vs* Nor,^#^*P*< 0.05 *vs* Ctrl. Note: CMECs cultured normally under normoxic conditions were set as Nor group. CMECs co-cultured with Treg cells for 48 hours were Ctrl group. Treg cells transfected with a lentivirus carrying a negative control were co-cultured with CMECs for 48 hours, referred to as the NC group. Treg cells transfected with a lentivirus carrying AREG were co-cultured with CMECs for 48 hours, referred to as the AREG group. CMECs co-cultured with Tregs transfected with a negative control vector were designated as the sh-NC group, while those co-cultured with Tregs in which AREG expression was silenced were designated as the sh-AREG group

To examine the effects of AREG⁺ Tregs on CMECs, we co-cultured Tregs overexpressing AREG with CMECs and assessed their proliferation, migration, and tube-forming ability under both normoxic and hypoxic conditions. The EdU assay revealed that CMECs co-cultured with AREG⁺ Tregs showed significantly increased proliferation (Fig. 4A and S6A). Cell cycle analysis confirmed a higher proportion of cells in the proliferative phase in CMECs exposed to AREG⁺ Tregs (Fig. 4B and S6B). Migration assays showed that AREG⁺ Tregs significantly enhanced CMEC migration (Fig. 4C and S6 C), and tube formation assays indicated that AREG⁺ Tregs promoted tube formation by CMECs (Fig. 4D and S6D). ELISA results further revealed that AREG, VEGF-A, and FGF were elevated in the co-culture system of CMECs and AREG⁺ Tregs (Fig. 4E and S6E), suggesting that AREG⁺ Tregs stimulate CMEC proliferation, migration, and angiogenesis.

To further evaluate the role of AREG in Tregs’ effects on angiogenesis, we inhibited AREG expression in Tregs by transfecting shRNA (S4A-S4B). Co-culture of AREG-inhibited Tregs with CMECs showed decreased CMEC proliferation, migration, and tube formation under both normoxic and hypoxic conditions (Fig. 4F–4J and S6 F-S6J). Inhibition of AREG in Tregs also led to a reduction in AREG, VEGF-A, and FGF levels in the co-culture system (Fig. 4K and S6K). These results collectively demonstrate that AREG is essential for Tregs to promote CMEC proliferation, migration, and angiogenesis under both normoxic and hypoxic conditions.

### AREG regulates FoxM1 to promote CMEC proliferation, migration, and tube formation

**Fig. 5:**
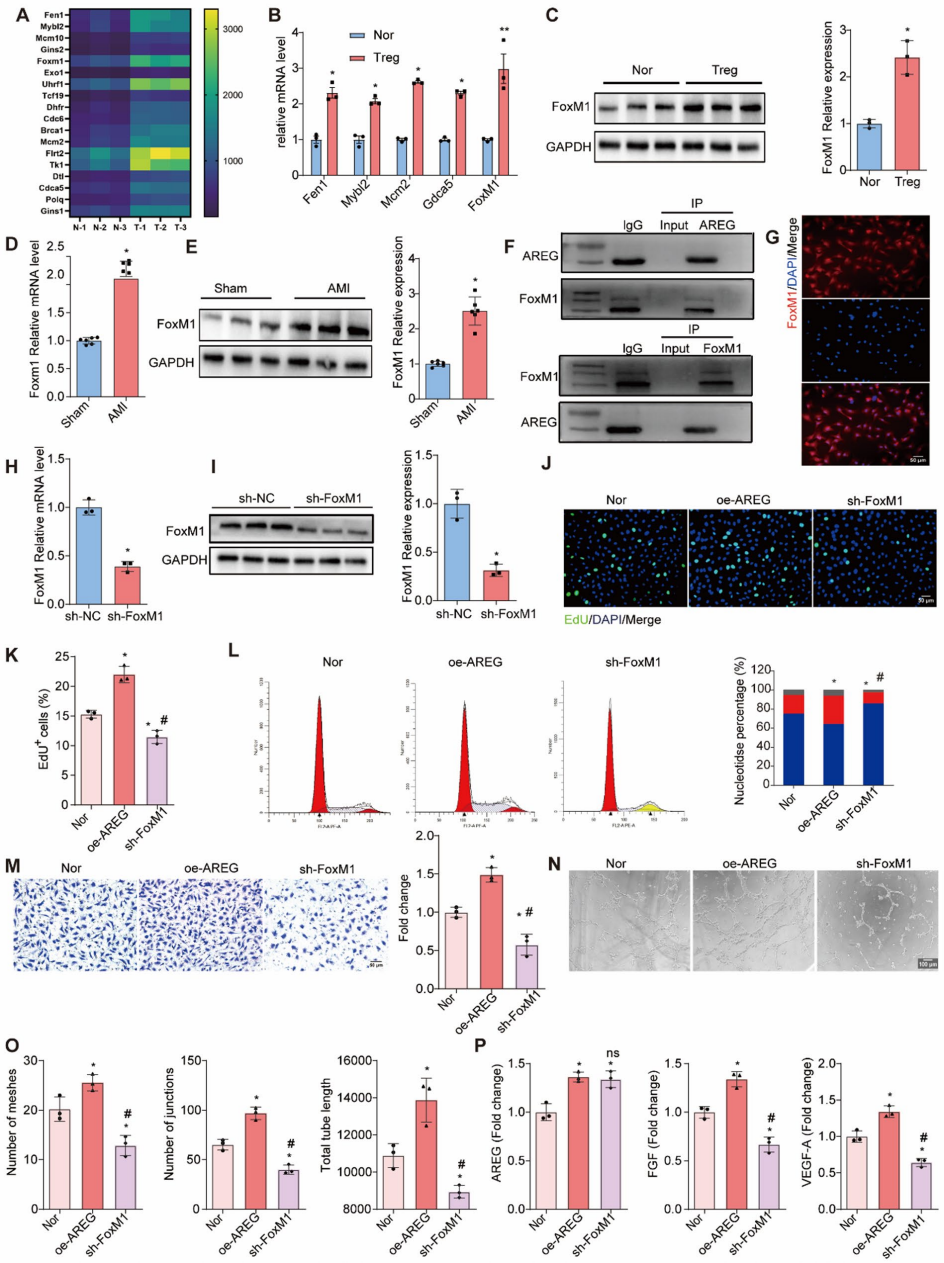
AREG target FoxM1 to regulate CMECs. **A **Heatmap of RNA-sequencing analysis showed that gene expression was significantly upregulated in CMECs of the AREG-overexpression Tregs (AREGgroup) compared with the Treg (Nor) group. For all statistical plots, the data are presented as the mean ± SEM. *p < 0.05 between the two indicated groups by 2-tailed Student’s *t*-test. **B **The relative mRNA level of Fen1, Myb12, Mcm10, Gins2, FoxM1 in Nor and Treg group. *n* = 3 per group.^*^P< 0.05 *vs* Nor. **C **FoxM1 protein levels in CMECs were quantified by Western blotting in Nor or Treg group. *n* = 3 per group.^*^P< 0.05 *vs* Nor. **D **The relative mRNA level of FoxM1 in Sham and AMI group. *n* = 3 per group.^*^P< 0.05 *vs* Nor. **E **FoxM1 protein levels in CMECs were quantified by Western blotting in Sham or AMI group. *n*= 3 per group.^*^P< 0.05 *vs* Nor. **F **Co-immunoprecipitation of AREG and FoxM1 in CMECs. **G **Immunofluorescence staining of FoxM1 in CMECs, showing nuclear localization. *n* = 3 per group.^*^P< 0.05 *vs* Nor.Scale bars, 50 mm. **H **qRT-PCR analysis of FoxM1 expression in CMECs transfected with si-NC and si-FoxM1. *n* = 3 per group.^*^P< 0.05 *vs* si-NC. **I **Western blot analysis of FoxM1 expression in CMECs transfected with si-NC and si-FoxM1. *n* = 3 per group.^*^P< 0.05 *vs* si-NC. **J **Representative EdU staining images of CMECs in the Nor, oe-AREG, and si-FoxM1 groups. **K **Quantification of EdU-positive cells in the Nor, oe-AREG, and si-FoxM1 groups. *n* = 3 per group.^*^P< 0.05 *vs* Nor,^#^*P*< 0.05 *vs* oe-AREG. **L **Flow cytometry analysis of the cell cycle distribution in the Nor, oe-AREG, and si-FoxM1 groups, with the corresponding bar graph displaying the percentage of cells in G0/G1, S, and G2/M phases. *n* = 3 per group.^*^P< 0.05 *vs* Nor,^#^*P*< 0.05 *vs* oe-AREG. **M **Migration assay results with quantification of migrated cells in the Nor, oe-AREG, and si-FoxM1 groups. *n* = 3 per group.^*^P< 0.05 *vs* Nor,^#^*P*< 0.05 *vs* oe-AREG. **N **Tube formation assay showing angiogenesis in the Nor, oe-AREG, and si-FoxM1 groups. **O **Quantification of the number of meshes, junctions, and total tube length in the Nor, oe-AREG, and si-FoxM1 groups. *n* = 3 per group.^*^P< 0.05 *vs* Nor,^#^*P*< 0.05 *vs* oe-AREG. **P **Relative expression levels of AREG, FGF, and VEGF-A in the Nor, oe-AREG, and si-FoxM1 groups. *n* = 3 per group.^*^P< 0.05 *vs* Nor,^#^*P*< 0.05 *vs* oe-AREG

To investigate the mechanism by which AREG in Tregs promotes endothelial angiogenesis, we performed RNA sequencing on CMECs co-cultured with Tregs or AREG-overexpressing Tregs. A total of 264 genes were up-regulated and 384 genes were down-regulated in CMECs after co-culture with AREG⁺ Tregs. The 18 most significantly up-regulated genes were selected for heat mapping (Fig. 5 A). Among these, FoxM1 was identified as the most prominently up-regulated gene (Fig. 5B), and its expression was validated at both mRNA and protein levels, showing significant upregulation in CMECs co-cultured with AREG-overexpressing Tregs (Fig. 5 C). Interestingly, FoxM1 expression was also significantly elevated in myocardial infarction tissue (Fig. 5D and E). Co-immunoprecipitation (Co-IP) experiments revealed a direct interaction between AREG and FoxM1 in infarcted heart tissue 7 days post-AMI (Fig. 5 F), and FoxM1 expression was also observed in CMECs (Fig. 5G).

To examine the role of FoxM1 in AREG⁺ Treg-mediated angiogenesis, CMECs were transfected with FoxM1-specific siRNA. Successful suppression of FoxM1 was confirmed by immunofluorescence, Western blot, and RT-qPCR (Fig. 5H and I). When FoxM1 was knocked down, the ability of AREG⁺ Tregs to promote CMEC proliferation, migration, and tube formation was significantly impaired (Fig. 5 J and O). Although the expression of AREG was unchanged in the co-culture system, inhibition of FoxM1 significantly suppressed VEGF-A and FGF levels (Fig. 5P). These results indicate that FoxM1 plays a critical role in AREG⁺ Treg-mediated angiogenesis, and its inhibition in CMECs dampens the angiogenic effects of AREG⁺ Tregs.

### AREG⁺Tregs promote post-myocardial infarction neovascularization via FoxM1

**Fig. 6:**
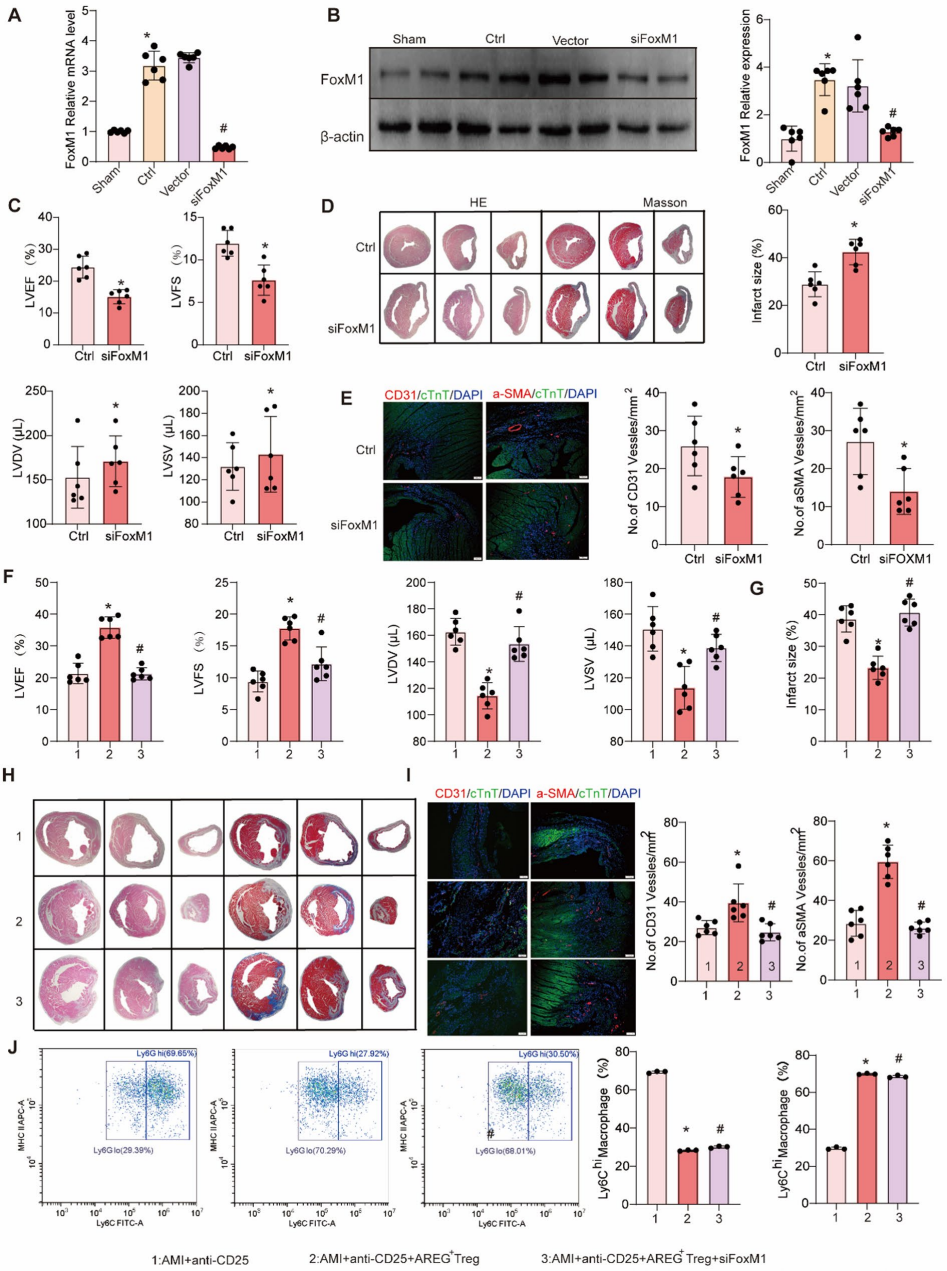
AREG⁺Tregs enhance neovascularization post-AMI through FoxM1. **A **qRT-PCR analysis of FoxM1 mRNA levels in the Sham, Ctrl, Vector, and siFoxM1 groups, with quantification showing a significant reduction in the siFoxM1 group. *n* = 6 per group.^*^P< 0.05 *vs* Sham,^#^*P*< 0.05 *vs* Ctrl. **B **Western blot analysis of FoxM1 expression in the Ctrl and siFoxM1 groups, with corresponding quantification of FoxM1 protein levels. *n* = 6 per group.^*^P< 0.05 *vs *Ctrl. **C **LVEF, LVFS, LVDV, and LVSV at day 28 post-AMI by echocardiography in Ctrl and siFoxM1 groups.*n* = 6 per group.^*^P< 0.05 *vs* Ctrl. **D **Representative HE and Masson staining of heart sections from Ctrl and siFoxM1 groups, showing increased infarct size in the siFoxM1 group, with quantification of infarct size percentage. *n* = 6 per group.^*^P< 0.05 *vs* Ctrl. **E **Immunofluorescence staining of CD31 and α-SMA in heart sections, indicating reduced capillary and arteriole density in the siFoxM1 group, with corresponding quantification of the number of vessels per mm². *n* = 6 per group.^*^P< 0.05 *vs* Ctrl. **F **LVEF, LVFS, LVDV, and LVSV at day 28 by echocardiography in AMI+anti-CD25, AMI+anti-CD25+AREG^+^Treg and AMI+anti-CD25+AREG^+^Treg+siFoxM1 group.*n* = 6 per group.^*^P< 0.05 *vs* AMI+anti-CD25.^#^*P*< 0.05 *vs* AMI+anti-CD25+AREG^+^Treg.**G **Quantification of infarct size across the three experimental groups. *n* = 6 per group. ^*^P< 0.05 *vs* AMI+anti-CD25.^#^*P*< 0.05 *vs* AMI+anti-CD25+AREG^+^Treg. **H **Representative HE and Masson staining images of heart sections in the three groups, demonstrating changes in myocardial tissue structure. **I **Immunofluorescence staining of CD31 and α-SMA in heart sections from the three experimental groups, with quantification showing enhanced angiogenesis in the AMI+anti-CD25+AREG^+^Treg group and reduced in AMI+anti-CD25+AREG^+^Treg+siFoxM1 group. *n* = 6 per group. ^*^P< 0.05 *vs* AMI+anti-CD25.^#^*P*< 0.05 *vs* AMI+anti-CD25+AREG^+^Treg. **J **Flow cytometry analysis showing Ly6C⁺ macrophage population percentages in the three experimental groups. *n* = 6 per group. ^*^P< 0.05 *vs* AMI+anti-CD25.^#^*P*< 0.05 *vs* AMI+anti-CD25+AREG^+^Treg

To investigate whether AREG⁺ Tregs mediate angiogenesis via FoxM1, we first confirmed that FoxM1 expression was elevated after AMI, and small interfering RNA (siRNA) significantly downregulated FoxM1 in myocardial tissue (Fig. 6A and B). Moreover, interfering with FoxM1 expression significantly impaired cardiac function, increased infarct size, and reduced neovascularization following myocardial infarction (Fig. 6 C and E). These findings align with previous studies linking FoxM1 to angiogenesis in endothelial cells (Wang et al. 2018).

Next, we used an anti-CD25 antibody to deplete Tregs in an AMI mouse model, followed by adoptive transfer of AREG⁺ Tregs and FoxM1 knockdown using siRNA (S5). Compared to the AMI + anti-CD25 group, treatment with AREG⁺ Tregs significantly enhanced capillarization in the infarct border zone (Fig. 6I), reduced infarct size (Fig. 6H), and improved left ventricular function (Fig. 6 F). However, FoxM1 knockdown reversed these beneficial effects of AREG⁺ Tregs on infarct size and cardiac function (Fig. 6F and I).

Additionally, we assessed the recruitment of monocytes to the infarct area, since Treg activation can induce anti-inflammatory monocyte differentiation. Our data showed that AREG⁺ Tregs increased the expression of anti-inflammatory Ly6 Clow monocytes and reparative macrophages (Fig. 6J). Importantly, FoxM1 knockdown did not affect monocyte recruitment, suggesting that the pro-angiogenic effects of AREG⁺ Tregs are independent of their impact on monocyte/macrophage recruitment.

## Discussion

**Fig. 7:**
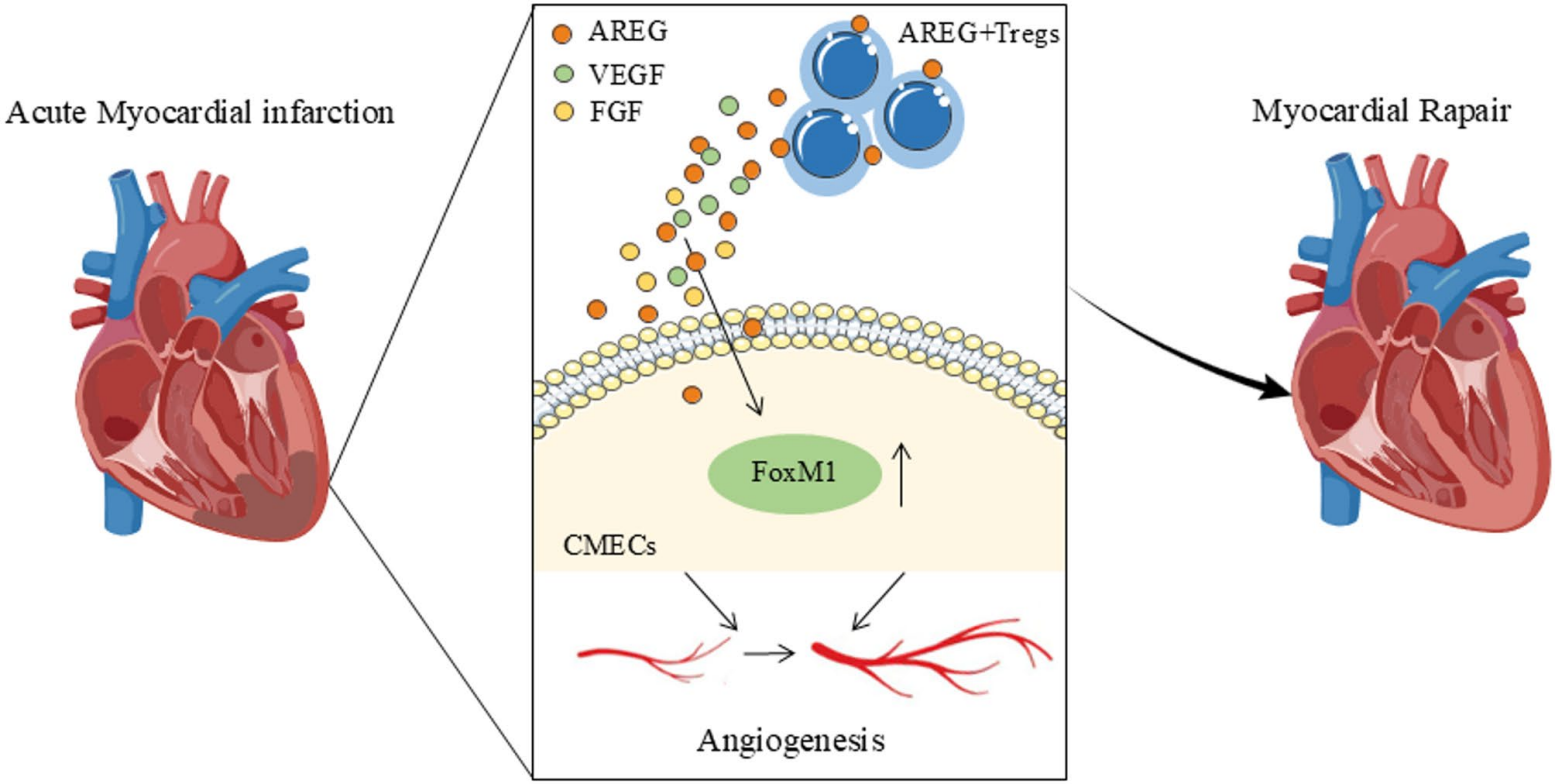
AREG-expressing Tregs promote angiogenesis following acute myocardial infarction by releasing AREG, VEGF, and FGF, hence improving cardiac functionality

Recent studies have demonstrated that Tregs exhibit significant plasticity and functional heterogeneity within different tissue microenvironments, beyond their initial immunosuppressive roles (Ito et al. 2019; Ali et al. 2017).Tregs expressing specific genes may serve as functional mediators of tissue repair post-acute myocardial infarction (AMI) (Zhuang et al. 2022). In this study, AREG expression was markedly upregulated following AMI in mice, and the number of AREG-positive Treg cells also increased post-AMI. In vivo expansion or adoptive transfer of exogenous Tregs significantly improved fibrosis area and cardiac function, but had no significant effect on neovascularization. Interestingly, AREG-modified Tregs further reduced the infarct size in mice, promoted neovascularization in the infarct zone, and significantly improved cardiac function. Additionally, based on transcriptomic data, we investigated the mechanism by which AREG^+^ Tregs enhance neovascularization and identified FoxM1 in endothelial cells as a potential target through which AREG promotes neovascularization (Fig. 7).

During acute myocardial infarction (AMI), various cytokines and angiogenic factors are activated, which not only serve as markers of myocardial injury and necrosis but also stimulate cardiac repair and promote post-AMI angiogenesis (Thiagarajan et al. 2017; Al-Kuraishy and Al-Gareeb 2020; Al-Kuraishy et al. 2016, 2021). This is pivotal in promoting myocardial infarction repair, as it not only decelerates the progression of myocardial infarction but also circumvents invasive surgical procedures and tissue/organ transplantation (Johnson et al. 2019). However, coronary microvascular endothelial cells (CMECs) lack sufficient angiogenic potential to effectively rebuild the vascular network, and clinical trials utilizing pro-angiogenic molecules have consistently failed (Kocijan et al. 2021). Therefore, identifying new approaches to restore blood flow in infarcted areas is essential to improving outcomes (Fung et al. 2020). Regulatory T cells, as a subset of T cells exerting immunosuppressive functions, infiltrate the myocardium post-infarction. Depletion of Treg cells leads to increased polarization of pro-inflammatory phenotype monocyte-derived macrophages, resulting in attenuation of inflammation and decreased expression of pro-healing factors post-AMI, leading to worsening of cardiac function, whereas in vivo expansion of Tregs can ameliorate cardiac remodeling post-AMI (Thiagarajan et al. 2017). In this study, we observed a significant increase in Tregs within the infarcted tissue, peaking on day 7 post-AMI. Previous studies have shown that anti-CD25 antibodies can partially deplete Tregs in vivo (He et al. 2024), while intraperitoneal injection of the IL-2/JES6-1 complex promotes Treg expansion (Tomala et al. 2021), both demonstrating good efficacy and safety (Bansal et al. 2019; Spangler et al. 2018). To further explore the role of Tregs in myocardial infarction (MI), we used anti-CD25 antibody-mediated partial depletion of Tregs. This approach led to reduced left ventricular ejection fraction and increased myocardial fibrosis post-MI. Conversely, stimulating Tregs with the IL-2/JES6-1 complex improved both cardiac function and fibrosis. Additionally, adoptive transfer of cultured Tregs promoted tissue repair in the injured myocardium. While evidence suggests that Tregs may be involved in angiogenesis under certain conditions (Fung et al. 2020; Leung et al. 2018), their specific role in post-AMI angiogenesis remains uncertain.and in a mouse model of lower limb ischemia induced by femoral artery ligation, Treg cells have been demonstrated to exert detrimental effects on neovascularization (Zouggari et al. 2009), and in a murine model of heart failure, phenotypically altered Treg cells can inhibit angiogenesis (Bansal et al. 2019). These findings underscore the need for further investigation into the role of Tregs in post-AMI angiogenesis to elucidate potential mechanisms. Our findings showed that neither Treg depletion nor expansion significantly influenced angiogenesis post-AMI. Intravenous injection of expanded Tregs did not enhance neovascularization in infarcted myocardium. Co-culture of Tregs with CMECs showed no impact on CMEC proliferation, migration, or tube formation, even under hypoxic conditions. These results may be attributed to the fact that under these experimental conditions, Treg cells did not exhibit a pro-angiogenic phenotype, and the co-culture system of Tregs with CMECs did not result in high levels of VEGF-A and FGF expression. Consequently, neither inhibition nor promotion of CMEC proliferation, migration, and tube formation occurred. It is evident that Tregs exhibit heterogeneity in cellular phenotype and function across different tissue microenvironments, as previously reported in the literature (Kumar et al. 2022; Lužnik et al. 2020). Interestingly, previous research has identified a subset of Tregs expressing amphiregulin (AREG) in myocardial tissue post-AMI (Zhuang et al. 2022), suggesting that AREG^+^ Tregs might play a distinct role in tissue repair and angiogenesis, warranting further investigation.

AREG is a transmembrane precursor protein and a ligand of the epidermal growth factor receptor (EGFR), initially identified as an epithelium-derived factor. However, it has since been shown to be expressed by activated immune cells in various inflammatory settings, including macrophages and Tregs (Zaiss et al. 2015; Sugita et al. 2021; Leung et al. 2018; Minutti et al. 2019). Previous studies have shown that AREG plays an important role in angiogenesis. In pulmonary arterial hypertension, the absence of AREG promotes pulmonary endothelial cell death and affects pulmonary vascular remodeling (Florentin et al. 2022).Additionally, Tregs also promote muscle regeneration and angiogenesis in ischemic limbs via AREG secretion (Liu et al. 2022). In this study, AREG expression in infarcted myocardial tissue peaked on day 7 post-AMI, and AREG-expressing Treg cells were most abundant at this time. While in vivo expansion of Tregs improved cardiac function, it did not significantly enhance angiogenesis. However, AREG-overexpressing Tregs improved cardiac function, reduced fibrosis, and promoted blood vessel formation in the infarct border zone. In vitro, AREG-modified Tregs enhanced or inhibited CMECs proliferation, migration, and tube formation, indicating that AREG^+^ Tregs play a crucial role in angiogenesis and myocardial repair following AMI.

Through mRNA sequencing and analysis, Forkhead box M1 (FoxM1), a transcription factor, was identified as significantly upregulated in CMECs co-cultured with AREG⁺ Treg cells. This suggests that FoxM1 may be one of the downstream targets through which AREG⁺ Treg cells promote CMEC proliferation and migration. FoxM1, a member of the forkhead transcription factor family, is known to mediate wound healing processes (Yang et al. 2022). Studies have shown that FoxM1 is a protective transcription factor in endothelial cells. Inhibition of its expression leads to impaired cell viability, increased apoptosis, elevated expression of pro-inflammatory cytokines, and diminished angiogenic activity (Zhang et al. 2020; Huang et al. 2019). FoxM1 is crucial for endothelial repair, as it reactivates endothelial regeneration, promotes vascular healing, reduces inflammation after pulmonary injury in septic mice, and improves survival in septic mice (Huang et al. 2023). Studies have also shown that FoxM1 is inhibited under copper ion stress in zebrafish embryos, which subsequently limits lymphangiogenesis and vasculogenesis. This suggests that FoxM1 regulates the formation of lymphatic and blood vessels during embryonic development (Tai et al. 2022). Importantly, AREG regulates the proliferation of keratinocytes by activating FoxM1-dependent transcriptional programs, thereby controlling G2/M phase progression and cytokinesis (Stoll et al. 2016). In our study, we found a protein interaction between AREG and FoxM1. Knockdown of FoxM1 in CMECs reduced the effect of AREG^+^ Tregs on CMEC proliferation, migration, and tube formation, and decreased VEGF-A and FGF secretion. In vivo, FoxM1 inhibition increased infarct size, reduced neovascularization, and worsened cardiac function, while reversing the reparative and angiogenic effects of AREG⁺ Tregs on myocardial infarction. These results suggest that AREG^+^ Tregs mediate their effects through FoxM1.Interestingly, while Tregs promote myocardial healing by recruiting anti-inflammatory macrophages (Gladow et al. 2024), FoxM1 inhibition did not affect macrophage recruitment in our model, indicating that AREG^+^ Tregs promote myocardial repair and angiogenesis independent of their impact on macrophages. Our findings highlight a distinct subset of cardiac Tregs expressing AREG, which improve myocardial fibrosis and cardiac function by enhancing neovascularization. These insights open new avenues for developing targeted therapies for myocardial infarction and heart diseases. Interestingly, statins hold an irreplaceable position in the treatment of acute coronary syndrome (ACS) patients, not only due to their lipid-lowering effects but also because of their modulation of inflammatory responses. Recent studies have shown that intensive statin therapy can enhance the expression of Tregs, increase the levels of anti-inflammatory cytokines such as IL-10 and TGF-β, and reduce the levels of pro-inflammatory cytokines like IFN-γ (Greca et al. 2023). These findings suggest that improving Treg expression and the immune regulatory balance they induce could provide clinical benefits for ACS patients.

However, translating our findings into clinical practice requires addressing several important considerations. For instance, further exploration of strategies to expand or modulate AREG^+^ Tregs in vivo is necessary. Additionally, the long-term safety and efficacy of AREG^+^ Treg-based therapies must be evaluated, including potential off-target effects and immune modulation. Clinical trials assessing the use of AREG^+ ^Tregs in combination with other therapeutic strategies, such as stem cell therapy or immune checkpoint inhibitors, could provide synergistic approaches to enhancing cardiac regeneration. Moreover, investigating the interaction of AREG^+^ Tregs with other immune cells and stromal cells in the infarcted myocardium will be crucial for optimizing their therapeutic potential.

### Limitations

In vivo research did not specifically deplete Tregs or AREG^+^ Tregs in mice; instead, we used anti-CD25 antibody for non-specific Treg cell depletion or IL-2/JES6-1 complex to expand Tregs in vivo, or intravenously injected cultured Tregs and AREG-overexpressing Tregs to assess their impact on post-myocardial infarction cardiac function, fibrosis, and angiogenesis. Additionally, this study did not conditionally knock out AREG in Tregs or FoxM1 in CMECs to validate the mechanism. However, by using adoptive transfer of AREG-overexpressing Tregs, the study investigated the effect of AREG^+^Tregs on post-myocardial infarction angiogenesis, and conducted rescue experiments by downregulating FoxM1 in myocardium or CMECs using lentivirus carrying small interfering RNA both in vivo and in vitro. Although our study demonstrates that AREG^+^ Tregs promote angiogenesis, we have not definitively established whether VEGF and FGF alone are sufficient to stimulate neovascularization or if their effects are enhanced through interaction with AREG. Future studies will focus on elucidating whether AREG directly induces angiogenesis or acts indirectly by modulating pro-angiogenic factors such as VEGF and FGF, and further investigate the role of the complete signaling cascade, including AREG and FoxM1, in post-myocardial infarction angiogenesis.

## Supplementary Information

Supplementary Material 1.

## Data Availability

No datasets were generated or analysed during the current study.

## Abbreviations

AMI: Acute myocardial infarction
AREG: Amphiregulin
α-SMA: α-smooth muscle actin
CMEC: Cardiac microvascular endothelial cell
VR: Ventricular remodeling
FoxP3^+^Tregs: Fork head box P3 regulatory T cells
LV: Left ventricle
Ly6 C: Lymphocyte antigen 6 complex, locus C
Ly6G: Lymphocyte antigen 6 complex, locus G
Treg: Regulatory T cell
LVEF: Left Ventricular Eject Fraction
LVFS: Left Ventricular Fraction Shortening
LVDV: Left Ventricular End-diastolic Volume
LVSV: Left Ventricular End-systolic Volume
FoxM1: Forkhead Box Protein M1
VEGFA: Vascular Endothelial Growth Factor
FGF: Fibroblast Growth Factor
