# Supplementary material for: AREG+ regulatory T cells mediating myocardial repair and neovascularization after myocardial infarction
Source: Mol Med. 2025 Jun 11;31:229. doi: 10.1186/s10020-025-01281-8 (PMC12153170; doi:10.1186/s10020-025-01281-8)
Supplement: Supplementary file 1 — Supplementary Material 1. [file 10020_2025_1281_MOESM1_ESM.docx]

**AREG^+^ regulatory T** **cells mediating myocardial repair and** [**neovascularization**](javascript:;) **after myocardial infarction**

Yan Wang^1,&^, Jiao Li^1,&^, Yu Zhang^1,&^, Pingping He^1^^, &^, Weiwei Liu^1^, Weirong Zeng^1^, Chaofu Li^1^, Yixuan Gao^1^, Yongchao Zhao^1^,Changyin Shen^1^, Wenming Chen^1^, Yunhang Li^2^, Ranzun Zhao^1*^, Bei Shi^1, *^

^1^Department of Cardiology, Affiliated Hospital of Zunyi Medical University, 563000

^2^Department of Cardiology, Guizhou Provincial Staff Hospital

^&^These authors contributed equally to this work.

^*^Correspondence: Ranzun Zhao, Bei Shi

Email: [shib@zmu.edu.cn](mailto:shib@zmu.edu.cn),

**Supplemental Materials and Methods**

**In Vivo Experimental Grouping**

To investigate the effects of Tregs on cardiac function and angiogenesis following acute myocardial infarction (AMI) in mice, the experiment was divided into several groups: (1) Sham group, (2) Control group (AMI only), (3) IgG group, where mice received isotype control antibody injections for three days prior to AMI induction, (4) anti-CD25 group, where Tregs were partially depleted with anti-CD25 before AMI, and (5) IL-2/JES6-1 group, where Tregs were expanded with IL-2/JES6-1 prior to AMI induction. Following AMI, Tregs were infused via tail vein injection in the presence or absence of anti-CD25-mediated Treg depletion to assess their contribution to post-AMI cardiac function and angiogenesis. Additionally, overexpression of AREG⁺ Tregs was used to evaluate their effects on cardiac function and angiogenesis after AMI. To further explore the role of FoxM1, the AMI + anti-CD25 group, AMI + anti-CD25 + AREG⁺ Tregs group, and AMI + anti-CD25 + AREG⁺ Tregs + siFoxM1 group were studied. This allowed the examination of how AREG⁺ Tregs affect cardiac function, angiogenesis, and monocyte-macrophage infiltration in Treg-depleted mice following FoxM1 inhibition in myocardial tissue.

**Mouse model of AMI**

A permanent ligation of the left anterior descending (LAD) artery or a sham procedure was performed on experimental animals according to a previously published protocol[1]. Mice were anesthetized with 1% isoflurane and maintained under general anesthesia. A 1.2 cm incision was made on the left chest, and the pectoralis major and external intercostal muscles were bluntly dissected to expose the heart. The LAD artery was ligated 2 mm below the left atrial appendage using a 7-0 suture [1]. Sham-operated mice underwent the same procedure without LAD ligation. The chest cavity was closed with a purse-string suture, and mice that did not survive the first 24 hours post-surgery were excluded from analysis. Electrocardiograms (ECG) were used to confirm successful coronary occlusion by detecting ST-segment elevation.and TTC staining, along with HE and Masson staining, confirmed myocardial fibrosis following the induction of AMI **(S1)**.


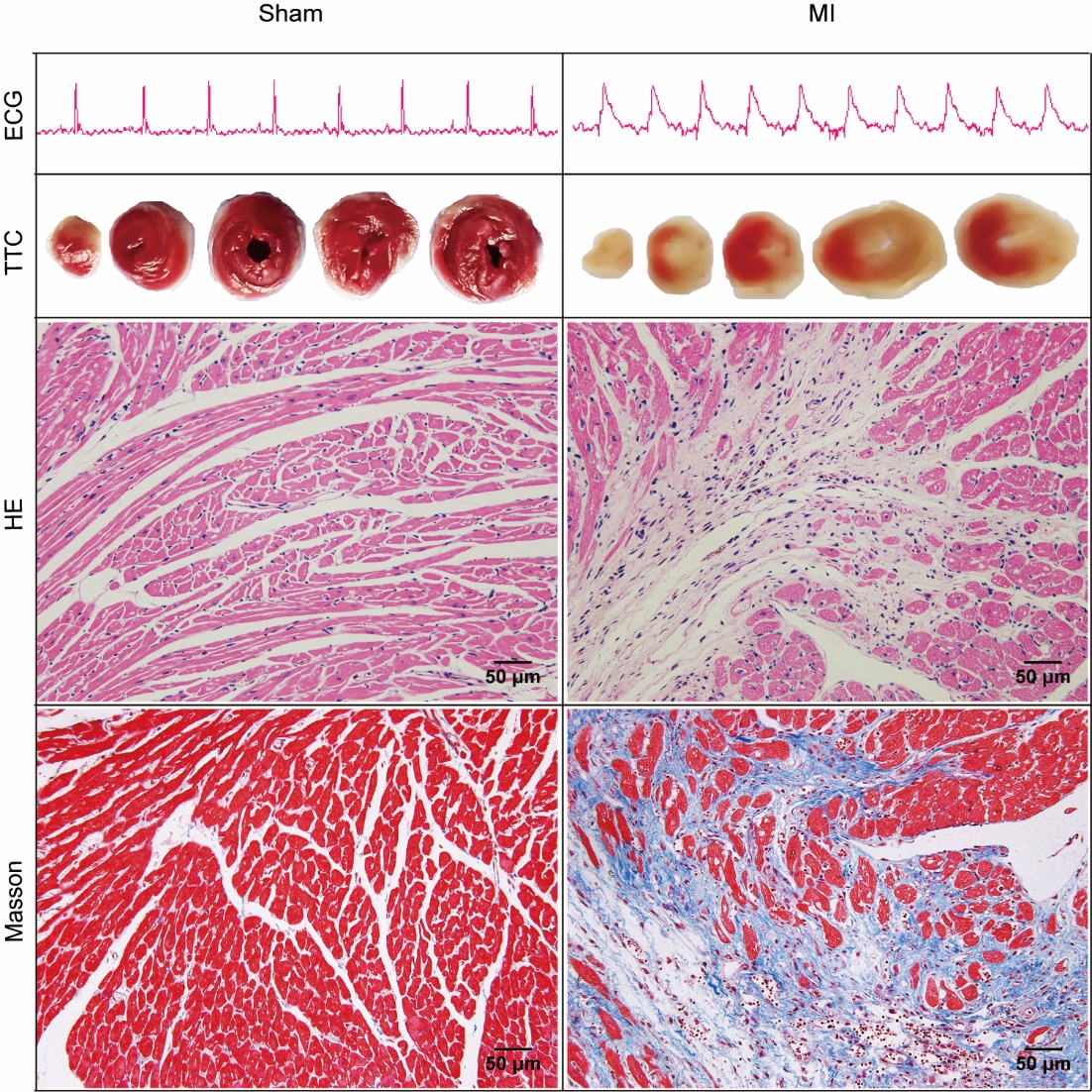


| **Supplement Figure 1:** A**cute myocardial infarction (AMI) model in mice.**  Representative images of electrocardiograms (ECG), TTC staining, and HE and Masson staining for both the sham-operated and acute myocardial infarction (AMI) groups in mice. |
| --- |

**Clearance and expansion of Treg cells in mice**

C57BL/6J mice used for the preparation of myocardial infarction model were injected intraperitoneally with anti-CD25 antibody 250μg per mouse 3 days before modeling to remove mouse treg cells in vivo[2], and the control group was injected with the same amount of isotype control IgG antibody **(S 2A)**.

C57BL/6J mice used to induce the myocardial infarction model were intraperitoneally injected with an IL-2/JES6-1 complex (5 μg JES6-1 + 1 μg IL-2 per mouse) daily for 3 days prior to surgery[3]. The control group received an equivalent dose of isotype control IgG antibody **(S 2B)**.

**Adoptive Transfer of Mouse Treg Cells**

Mouse Treg cells were collected from in vitro cultures and resuspended in PBS for counting. The cells were centrifuged at 1500 rpm for 5 minutes, and the supernatant was discarded. A total of 1 × 10^⁶ Treg cells were resuspended in 200 μL PBS and injected via the tail vein on the day of AMI model induction. In the control group, 200 μL of PBS was injected via the tail vein without Treg cells **(S 2C)**.


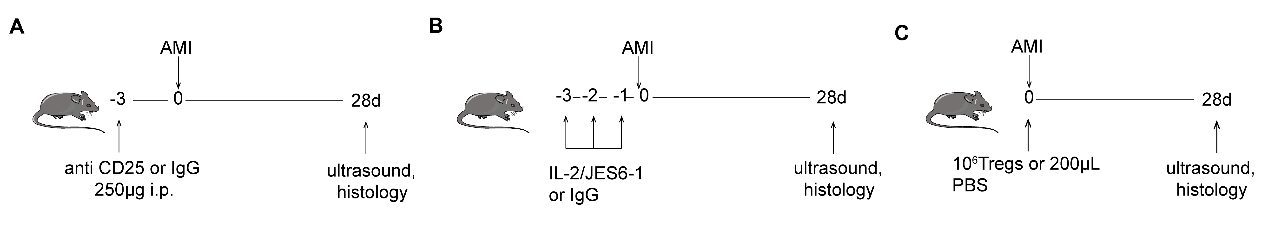


| **Supplement Figure 2:** Schematic representation of the experimental design. **A.** Mice were intraperitoneally injected with anti-CD25 (250 μg/mouse) or isotype control IgG three days prior to acute myocardial infarction (AMI) induction. Ultrasound and histology were performe  d at day 28 post-AMI. **B.**Mice received IL-2/JES6-1 complex (6 μg/mouse) or isotype control IgG intraperitoneally for three consecutive days before AMI. Ultrasound and histological assessments were conducted 28 days after AMI.**C.** On the day of MI induction (Day 0), 1 × 10⁶ Treg cells or 200 μL of PBS were administered intravenously (i.v.) via the tail vein. Cardiac function and tissue remodeling were assessed by ultrasound and histological analysis 28 days post-AMI. |
| --- |

**Flow cytometric analysis**

Single-cell suspensions were prepared as previously described with slight modifications[4]. Briefly, mice were deeply anesthetized and intracardially perfused with 40 ml of ice-cold PBS to remove blood cells. Hearts were then dissected, minced, and enzymatically digested in Hanks’ balanced salt solution (HBSS, Sigma-Aldrich) containing 1 mg/ml type II collagenase, 100 U/ml elastase (both from Worthington Biochemical Corporation), and 100 U/ml DNase I (Sigma-Aldrich) for 1.5 hours at 37°C with gentle agitation. The digested tissue was triturated and passed through a 70 μm cell strainer (BD Falcon), followed by centrifugation (15 min, 500 × g, 4°C). Post-erythrocyte lysis, cells were washed with RPMI-1640 medium and counted for further analysis. To block nonspecific binding to Fcγ receptors, cells were incubated with anti-CD16/32 antibody (BD Bioscience) at 4°C for 5 min. The cells were then stained with the following antibodies for 20 min at 4°C: CD45-APC-Cy7 (Biolegend , #103114), FITC anti-mouse CD4(Biolegend, #100510), and PE anti-mouse Foxp3 (Biolegend, #126404).CD11b (eBioscience, #25-0112-82), Ly6G (eBioscience, #145931), F4/80 (eBioscience, #17-4801-82), Ly6C (BioLegend, #128018), MHCII (BioLegend, #107618) (**S3**).Flow cytometric analysis was performed using a FACSAria (BD Biosciences), and data were analyzed with FlowJo software (Tree Star)[5].

**
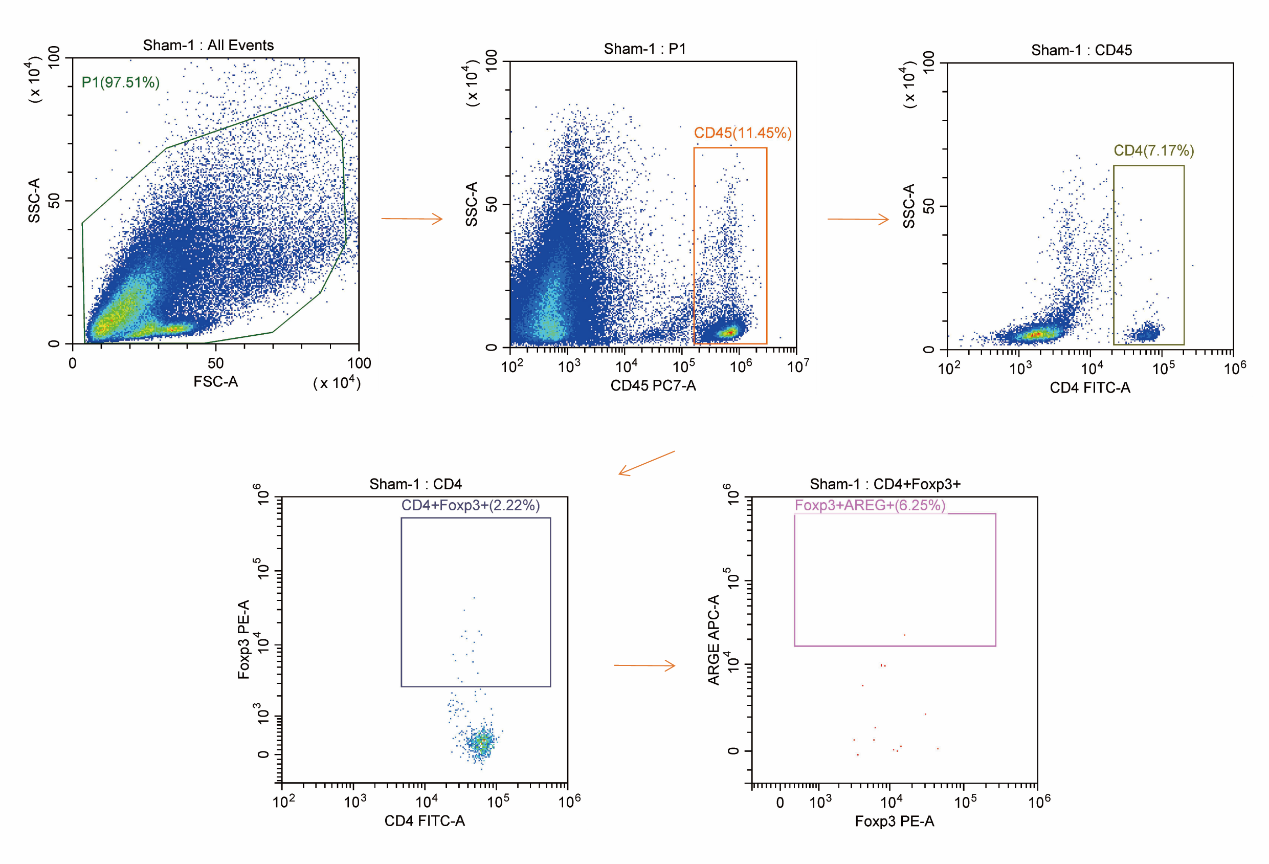
**

| **Supplement Figure 3: The gating strategy for sorting Tregs from heart tissue homogenates.** Tregs were identified as CD45^+^CD4^+^foxp3^+^.AREG^+^Tregs were identified as CD45^+^CD4^+^foxp3 ^+^ AREG^+^. |
| --- |

**Transthoracic echocardiography**

Echocardiography was conducted on day 28 post-MI using a Vevo 2100 imaging system equipped with an MS400 transducer (VisualSonics, Toronto, Canada). Mice were sedated with 2% isoflurane and placed supine on a temperature-controlled platform. Chest hair was removed with depilatory cream, and heart rate and body temperature were continuously monitored. Left parasternal long-axis views were captured to assess ejection fraction, and basal short-axis views were used to calculate fractional shortening. Three consecutive cardiac cycles were analyzed and averaged for each measurement [6].

**Histologic analysis**

Mice were euthanized via cervical dislocation, and their hearts were excised. The hearts were immediately perfused with normal saline, fixed in 4% paraformaldehyde (Servicebio, Wuhan, China), and placed on an orbital shaker overnight at 4°C. The tissues were then dehydrated, embedded in paraffin, and sectioned into 5 μm slices[7]. Hematoxylin-eosin and Masson’s trichrome staining (Solarbio, Beijing, China) were performed according to the manufacturer’s protocols. Fibrotic areas were visualized under a microscope (Olympus, Tokyo, Japan), and images were analyzed using ImagePro Plus software.

**Immunofluorescence staining**

The paraffin sections were deparaffinized and subjected to antigen retrieval using citrate buffer. Samples were blocked with goat serum at 25°C for 1 hour, followed by incubation with primary antibodies: mouse monoclonal anti-cardiac troponin T (Abcam, ab8295, 1 µg/ml), rabbit polyclonal anti-CD31 (Proteintech Group, 28083-1-AP, 1:20), and rabbit polyclonal anti-alpha smooth muscle actin (Abcam, ab124964, 1:250) at 4°C overnight. Cells were also incubated with primary antibodies including rabbit polyclonal anti-CD31 (Proteintech, 28083-1-AP, 1:500) and anti-vWF (Bosterbio, PB9273, 1:250) under the same conditions.After primary antibody incubation, sections and cells were treated with Alexa Fluor-conjugated secondary antibodies for 1 hour at room temperature, followed by DAPI counterstaining (Beyotime Biotechnology, Jiangsu, China). Images were captured using an Olympus BX61 microscope and analyzed with ImagePro Plus software[8].

**Real-time quantitative PCR**

Total RNA from the heart or CMECs was extracted using RNAiso Plus (Takara Bio, Shiga, Japan), according to the manufacturer’s instructions. The concentration of the isolated RNA was determined using an Ultra-Micro spectrophotometer (Implen, Munich, Germany). cDNA was synthesized using PrimeScript^™^ RT Master Mix (Takara Bio, Shiga, Japan). A quantitative real-time PCR assay was performed using the Fast SYBR Green Master Mix on the ViiA 7 Real-Time PCR system (Bio-rad, Hercules, CA, USA). The mRNA level of each sample was normalized to that of β-actin. The data were computed using the 2^−ΔCt^ method. The primer sequences (Sangon Biotech, Shanghai, China) used in the RT-qPCR assay were as follows:

| **Gene** | **Primer sequence** |
| --- | --- |
| *β-actin* | *Forward 5’-GCTATGCTCTCCCTCACG-3’*  *Reverse 5’-ACGCACGATTTCCCTCT-3’* |
| *AREG* | *Forward 5’-TTTGGTGAACGGTGTGG-3’*  *Reverse 5’-AATAGCTGCGAGGATGATG-3’* |
| *Foxm1* | *Reverse 5’-AATAGCTGCGAGGATGATG-3’*  *Reverse 5’-AATAGCTGCGAGGATGATG-3’* |
| *Fen1* | Forward 5’-AGAGCATCCGTGGCATT-3’  Forward 5’-AGAGCATCCGTGGCATT-3’ |
| *Mybl2* | Forward 5’-GTACGCTTCGCCCCTTC-3’  Forward 5’-GTACGCTTCGCCCCTTC-3’ |
| *Mcm10* | Forward 5’-GCCACTCTCTTTGGTGATG-3’  Forward 5’-GCCACTCTCTTTGGTGATG-3’ |
| *Gins2* | Forward 5’-TGGATGTGGAGAAACTGGA-3’  Reverse 5’-TGCTTTGGGGATGTTGTC-3 |

**Western blotting analysis**

Total protein from heart-derived Tregs or CMECs was extracted using RIPA lysis buffer supplemented with phosphatase and protease inhibitors (Beyotime Biotechnology, Jiangsu, China). Protein concentrations were determined using the BCA Protein Assay Kit (Solarbio, Beijing, China). Equal amounts of protein (20 μg per lane) were separated on 10% SDS-PAGE gels (Epizyme Biomedical Technology, Shanghai, China) and transferred to PVDF membranes. Membranes were blocked with 5% nonfat dry milk for 1 hour at 27°C, followed by overnight incubation with primary antibodies at 4°C. The primary antibodies included AREG (Proteintech, 16036-1-AP, 1:1000, Chicago, IL, USA) and FoxM1 (Proteintech, 13147-1-AP, 1:1000, Chicago, IL, USA).The membranes were then incubated with appropriate secondary antibodies for 1 hour at room temperature. Protein bands were visualized using ECL FemtoLight Oxidant (Epizyme Biomedical Technology, Shanghai, China), and band intensity was quantified using ImageJ software.

**Enzyme-linked immunosorbent assay (ELISA)**

The levels of VEGF-A, fibroblast growth factor (FGF), and AREG in the culture supernatants were determined using ELISA kits according to the manufacturer’s instructions (MEIMIAN, Jiangsu, China).

**Isolation and culture of Treg cells**

C57BL/6J mice (6–8 weeks old) were euthanized via cervical dislocation and disinfected with 75% ethanol for 5 minutes. Under aseptic conditions, the spleen was removed, rinsed with pre-chilled PBS, and the surrounding adipose tissue was carefully trimmed. The spleen was minced in pre-chilled PBS, and the cell suspension was obtained by filtering through a 400-mesh sieve. Erythrocytes were lysed by adding 2–3 mL of erythrocyte lysis buffer for 5 minutes, and the reaction was terminated by adding 2–3 mL of complete medium (10% FBS). The lysate was passed through another filter to yield a single-cell suspension, which was collected in a 15 mL centrifuge tube and centrifuged at 600g for 5 minutes. The cell pellet was resuspended in 5 mL of buffer, and cell counting was performed.A CD4^+^CD25^+^ regulatory T cell isolation kit was used to sort Tregs. Cells were resuspended in 40 μL buffer per 10⁷ cells, followed by the addition of 10 μL of CD4^+^CD25^+^ regulatory T cell Biotin-Antibody Cocktail and 38 μL buffer per 10⁷ cells. After mixing, 20 μL of Anti-Biotin MicroBeads and 2 μL of CD25-PE antibody were added per 10⁷ cells. The mixture was incubated at 2–8°C for 15 minutes. Sorting was performed using an MS column placed on a magnetic stand. After rinsing with 2 mL buffer, the sorted CD4+ T cell suspension was collected. The cells were centrifuged at 600g for 5 minutes, and the supernatant was removed. The pellet was resuspended in 90 μL buffer with 10 μL anti-PE magnetic beads, incubated at 2–8°C for 15 minutes, and sorted again using the same method.After the final sort, CD4+CD25+ T cells were collected and resuspended in RPMI-1640 medium containing 10% FBS, 1% penicillin-streptomycin, 2% glutamine, 8 μg/mL anti-CD3 antibody, 4 μg/mL anti-CD28 antibody, 10 ng/mL recombinant IL-2, and 10 ng/mL recombinant TGF-β1. Cells were then cultured in 6-well plates and incubated at 37°C in a 5% CO2 atmosphere[9].

**Cardiac microvascular endothelial cell (CMEC) culture**

Newborn mice (<3 days old) were sterilized with 75% ethanol for 10 seconds. The left ventricle was then isolated, and excess tissue was removed. The myocardial tissue was washed three times with pre-chilled PBS and briefly soaked in 75% ethanol for 15 seconds. After washing, the tissue was cut into approximately 1 × 1 × 1 mm blocks and evenly distributed in a 25 cm² culture flask containing 1 mL of fetal bovine serum. The flask was incubated at 37°C with 5% CO₂ for 4 hours. Once the tissue blocks adhered, 2 mL of endothelial cell complete medium (containing 10% fetal bovine serum, 1% penicillin, and 1% heparin) was gently added, and incubation continued under the same conditions. After approximately 7 days, paving stone-like monolayer cells were observed. Cells were subcultured at 80–90% confluence, and CMECs within three passages were used for the experiments[10]. For hypoxia treatment, the cells were placed in a hypoxia chamber (Thermo Fisher Scientific, Waltham, MA, USA) and subjected to 1% O2, 5% CO2, and 94% N2 for 24 hours at 37°C. Control CMECs were maintained under normoxic conditions (21% O2, 5% CO2) in a standard incubator.After the treatment, the cells were harvested for subsequent assays, including cell viability, proliferation, and protein expression analysis [11].

**EdU-labeling experiments**

The proliferation of CMECs was assessed using the EdU proliferation assay kit (RIBOBIO, C10310, Guangzhou, China) according to the manufacturer's instructions. Briefly, 2.5 × 10^5 CMECs were incubated with EdU for 2 hours, followed by fixation in 4% paraformaldehyde for 30 minutes at room temperature. The cells were then stained with Apollo for 30 minutes and counterstained with Hoechst 33342 to label the nuclei. Fluorescence microscopy (Olympus, Tokyo, Japan) was used to visualize and capture images of the stained cells.

**Flow cytometric analysis of cell cycle**

The cell cycle of CMECs was assessed using flow cytometry with a cell cycle detection kit, following the manufacturer’s protocol (4A Biotech, FXP0211, Beijing, China). Briefly, 1×10^6 CMECs were harvested and fixed in 70% ethanol at 4°C for 24 hours. Cells were then stained with propidium iodide (PI) solution and incubated at 37°C for 30 minutes. Flow cytometric analysis was performed using a Guava flow cytometer (Millipore, MA, USA).

**Cell migration assay**

CMECs (2 × 10^4 cells/well) were seeded into the upper chamber of an 8 μm pore-sized Transwell insert (Corning 3422, Corning, NY, USA), while Tregs (1 × 106cells/well) were seeded in the lower chamber [12]. The co-culture was maintained in a CO₂ incubator for 12 hours. After incubation, the inserts were removed, washed twice with PBS, and fixed in 4% paraformaldehyde for 15 minutes. The chambers were then washed 2–3 times with PBS, stained with crystal violet for 10 minutes, and rinsed again with PBS. Once air-dried, the migrated cells were visualized under a microscope, and images were captured from five randomly selected fields. Migration was quantified using ImageJ software.

**CMEC tube formation assay**

CMECs were seeded on Matrigel Matrix (Corning (354230), Corning, NY, USA) in μ-Slide angiogenesis slides (ibidi (81506), Martin Reid, Germany) at a density of 2 × 10^4 cells/well, for 4 h, according to the manufacturer’s instructions. Imaging was performed using an Olympus BX61 microscope. One field at 50× magnification was used for each well. Quantification was carried out by counting the number of junctions and meshes and the total tube length per 50× image by imageJ.

**Overexpression and inhibition of AREG in Tregs**

Lentiviruses overexpressing AREG or containing an empty vector, as well as AREG interference or control lentiviruses, were synthesized by HanHeng Biotechnology (Shanghai, China). Cell transfection was performed according to the manufacturer's protocol. Briefly, cultured suspension Tregs were counted and centrifuged at 600g for 5 minutes to collect the cell pellet. The cell number was adjusted to 1 × 10^5 CMECs in a culture flask.Based on preliminary experiments, an MOI of 200 was selected. The required viral volume was calculated according to cell number and viral titer, then added to the Treg culture medium. Tregs were resuspended in the virus-containing medium, transferred to a 6-well plate, and centrifuged at 200g for 1 hour using a flat-angle rotor. Cells were then incubated at 37°C with 5% CO₂. After 48 hours, infection efficiency was confirmed by Western blot (WB) and RT-qPCR[13].


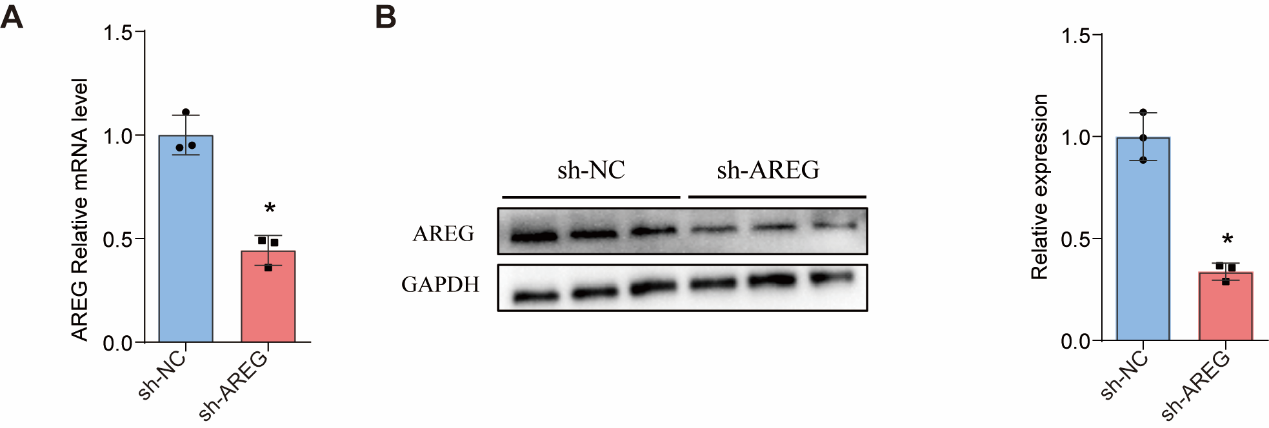


| **Supplement Figure 4: Expression of AREG in Tregs after interference. A .** The relative mRNA level of AREG in sh-NC and sh-AREG group. n = 6 per group. ^*^*P*< 0.05 *vs* sh-NC.**B.**AREG protein levels in the infarcted myocardium were quantified by Western blotting at sh-NC and sh-AREG group. |
| --- |

**Inhibition of FoxM1 in CMECs**

RNA interference (shRNA) in CMECs (1 × 10^5 cells/well) was conducted using 21-nucleotide RNA duplexes. Four FoxM1-targeting shRNAs were synthesized by Shanghai GenePharma Co. (Shanghai, China): FoxM1-homo-461 (5′-GCT GGG ATC AAG ATT ATT AAC-3′), FoxM1-homo-579 (5′-GCA GTA GTG GGC CCA ACA AAT-3′), FoxM1-homo-1044 (5′-GGA AGC GCA TGA CTT TGA AAG-3′), and FoxM1-homo-1822 (5′-GGA AAT GCT TGT GAT TCA ACA-3′). A non-targeting control shRNA (FoxM1-homo-NC, 5′-ACT ACC GTT GTT ATA GGT G-3′) labeled with FAM fluorophore was used to monitor transfection efficiency. The shRNA duplexes were introduced using Lipofectamine 2000 (Invitrogen, Carlsbad, CA, USA) following the manufacturer’s protocol.


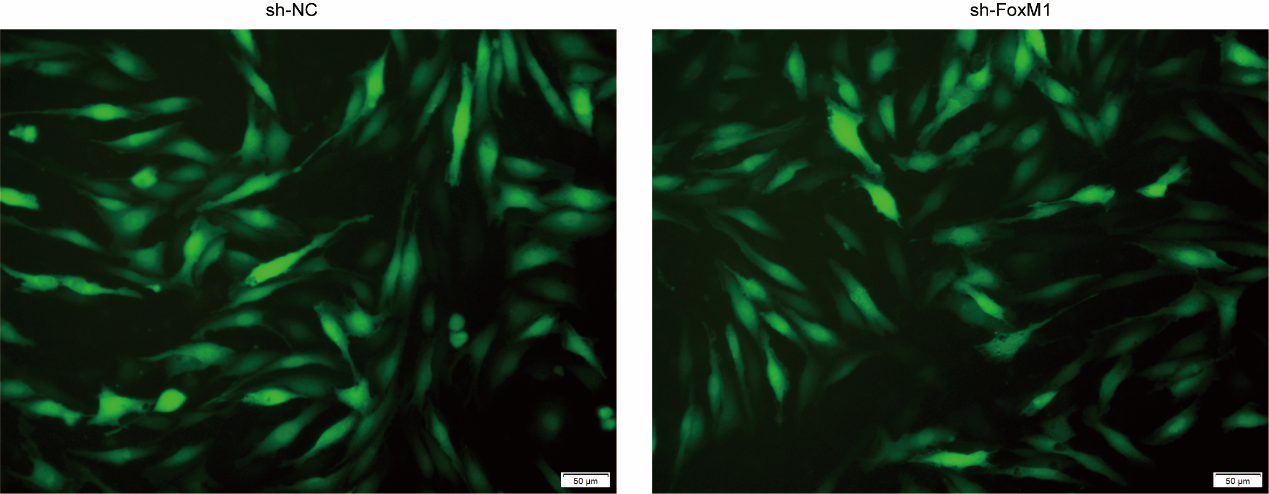


| **Supplement Figure 5: The expression of green fluorescent protein was detected by immunofluorescence.** Representative fluorescence images demonstrate the localization and expression levels of EGFP within the cells, confirming successful transfection. |
| --- |

**FoxM1 inhibition *in vivo***

The inhibition of FoxM1 expression in the myocardium was performed based on previous studies with slight modifications. Specifically, the procedure was initiated immediately following myocardial infarction (MI) in mice.100 μg of adenovirus (a titer of 1×10^9^v.g/ml containing an empty vector or the target gene, designed by PackGene Biotech) was injected via a micro-infusion pump *in situ* at multiple points in the infarct border zone below the ligature line(5 points in total, 20 μg per injection per point) into left ventricular anterior wall with six mice for each injection[14].


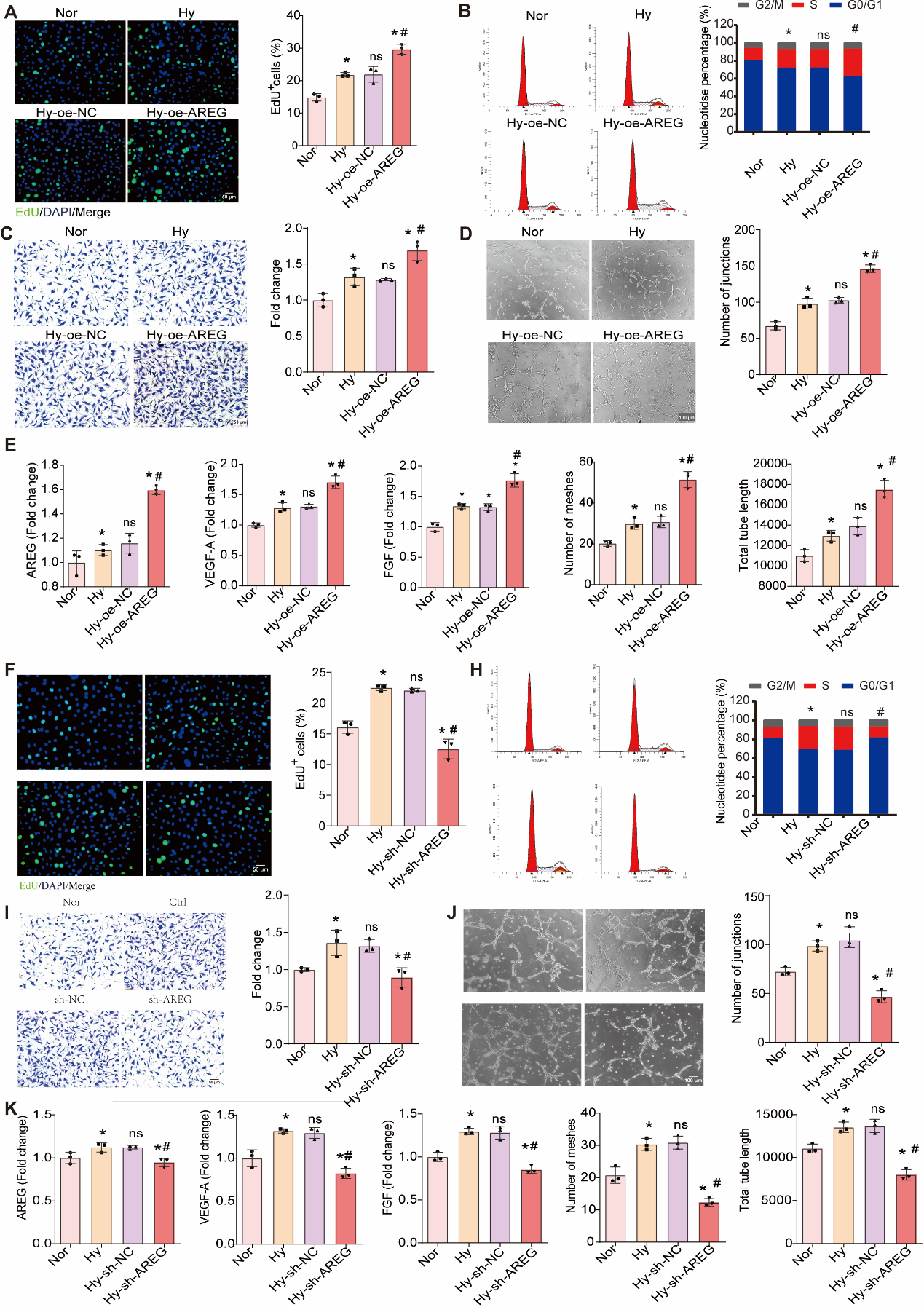


| **Supplement Figure 6.** **AREG+ Tregs mediate CMEC proliferation, migration, and tube formation under hypoxic conditions.**  **A.** Representative EdU staining images of CMECs,quantification of EdU-positive cells is shown on the right. n = 3 per group. ^*^*P*< 0.05 *vs* Nor, ^#^*P*< 0.05 *vs* Hy.  **B.** Flow cytometry analysis of the cell cycle in CEMCs in the Nor, Ctrl,oe-NC,oe-AREG groups. The proportion of cells in G0/G1, S, and G2/M phases is displayed in the accompanying bar graph. n = 3 per group. ^*^*P*< 0.05 *vs* Nor, ^#^*P*< 0.05 *vs* Hy.  **C.** Representative migration assay results (crystal violet staining) with corresponding quantification of fold change in migrated cells. ^*^*P*< 0.05 *vs* Nor, ^#^*P*< 0.05 *vs* Hy.  **D and E.** Tube formation assay illustrating the effects of AREG overexpression on angiogenesis, with quantification of the number of junctions ,meshs and total tuble length. ^*^*P*< 0.05 *vs* Nor, ^#^*P*< 0.05 *vs* Hy.  **F.** Expression of AREG, VEGF-A, and FGF in CMECs co-cultured with Tregs under the specified conditions. ^*^*P*< 0.05 *vs* Nor, ^#^*P*< 0.05 *vs* Hy.  **G.** Representative EdU staining images of CMECs in the Nor, Ctrl,sh-NC,sh-AREG groups,and quantification of EdU-positive cells is shown on the right. n = 3 per group. ^*^*P*< 0.05 *vs* Nor, ^#^*P*< 0.05 *vs* Hy.  **H.** Flow cytometry analysis of the cell cycle in CEMCs in the Nor, Ctrl,sh-NC,sh-AREG groups. The proportion of cells in G0/G1, S, and G2/M phases is displayed in the accompanying bar graph. n = 3 per group. ^*^*P*< 0.05 *vs* Nor, ^#^*P*< 0.05 *vs* Hy.  **I.** Representative migration assay results (crystal violet staining) with corresponding quantification of fold change in migrated cells. ^*^*P*< 0.05 *vs* Nor, ^#^*P*< 0.05 *vs* Hy.  **J and K.** Tube formation assay illustrating the effects of CMECs in the Nor, Ctrl,sh-NC,sh-AREG groups,with quantification of the number of junctions ,meshs and total tuble length. ^*^*P*< 0.05 *vs* Nor, ^#^*P*< 0.05 *vs* Hy.  **L.** Expression of AREG, VEGF-A, and FGF in CMECs co-cultured with Tregs under the specified conditions. ^*^*P*< 0.05 *vs* Nor, ^#^*P*< 0.05 *vs* Hy.  Note:CMECs cultured normally were set as Nor group. CMECs cultured under hypoxia for 24 hours were set as Hypoxia (Hy) group. Treg cells transfected with a lentivirus carrying a negative control were co-cultured with hypoxia-treated CMECs for 48 hours, referred to as the Hy-OE-NC group. Treg cells transduced with a lentivirus carrying AREG were co-cultured with hypoxia-treated CMECs for 48 hours, referred to as the Hy-OE-AREG group. Under the same hypoxic conditions,CMECs co-cultured with Tregs transfected with a negative control vector were designated as the sh-NC group, while those co-cultured with Tregs in which AREG expression was silenced were designated as the sh-AREG group. |
| --- |

**Relevant studies were selected based on the following criteria**

Studies that directly addressed Tregs, myocardial infarction (AMI), angiogenesis, and the role of FoxM1 were prioritized. Only articles that were pertinent to the specific cellular and molecular mechanisms of Treg function in cardiovascular diseases were included.And recent publications (preferably within the last 5years) were emphasized to ensure the inclusion of up-to-date knowledge and techniques.In addition, more consideration should be given to peer-reviewed journals with established impact factors or high citation rates in the field were considered. Studies using experimental models similar to those in our research (e.g., mouse models of AMI, adoptive transfer of Tregs, or FoxM1 inhibition) were prioritized for better comparability.

**References**

1. Zamilpa R, Zhang J, Chiao YA, de Castro Bras LE, Halade GV, Ma Y, Hacker SO and Lindsey ML (2013) Cardiac wound healing post-myocardial infarction: a novel method to target extracellular matrix remodeling in the left ventricle. Methods Mol Biol 1037:313-24. doi: 10.1007/978-1-62703-505-7_18

2. Albakri Aa, Coronel J, Pinos I, Blanco A, McQueen P, Molina D, Sim J, Fisher EA and Amengual J (2024) β-Carotene accelerates the resolution of atherosclerosis in mice. eLife 12. doi: 10.7554/eLife.87430

3. Tomala J, Weberova P, Tomalova B, Jiraskova Zakostelska Z, Sivak L, Kovarova J, Kovar M (2021) IL-2/JES6-1 mAb complexes dramatically increase sensitivity to LPS through IFN-γ production by CD25+Foxp3- T cells. eLife, 10. doi.org/10.7554/eLife.62432

4. Zhang Y, Wang Y, Li J, Li C, Liu W, Long X, Wang Z, Zhao R, Ge J and Shi B (2023) ANNEXIN A2 FACILITATES NEOVASCULARIZATION TO PROTECT AGAINST MYOCARDIAL INFARCTION INJURY VIA INTERACTING WITH MACROPHAGE YAP AND ENDOTHELIAL INTEGRIN Beta3. Shock 60:573-584. doi: 10.1097/SHK.0000000000002198

5. DeBerge M, Glinton K, Subramanian M, Wilsbacher LD, Rothlin CV, Tabas I and Thorp EB (2021) Macrophage AXL receptor tyrosine kinase inflames the heart after reperfused myocardial infarction. Journal of Clinical Investigation 131. doi: 10.1172/jci139576

6. Lichtenauer M, Mildner M, Baumgartner A, Hasun M, Werba G, Beer L, Altmann P, Roth G, Gyongyosi M, Podesser BK and Ankersmit HJ (2011) Intravenous and intramyocardial injection of apoptotic white blood cell suspensions prevents ventricular remodelling by increasing elastin expression in cardiac scar tissue after myocardial infarction. Basic Res Cardiol 106:645-55. doi: 10.1007/s00395-011-0173-0

7. Enzan N, Matsushima S, Ikeda S, Okabe K, Ishikita A, Yamamoto T, Sada M, Miyake R, Tsutsui Y, Nishimura R, Toyohara T, Ikeda Y, Shojima Y, Miyamoto HD, Tadokoro T, Ikeda M, Abe K, Ide T, Kinugawa S and Tsutsui H (2023) ZBP1 Protects Against mtDNA-Induced Myocardial Inflammation in Failing Hearts. Circ Res 132:1110-1126. doi: 10.1161/CIRCRESAHA.122.322227

8. Chen B, Huang S, Su Y, Wu YJ, Hanna A, Brickshawana A, Graff J and Frangogiannis NG (2019) Macrophage Smad3 Protects the Infarcted Heart, Stimulating Phagocytosis and Regulating Inflammation. Circ Res 125:55-70. doi: 10.1161/CIRCRESAHA.119.315069

9. Alshoubaki YK, Nayer B, Lu YZ, Salimova E, Lau SN, Tan JL, Amann-Zalcenstein D, Hickey PF, Del Monte-Nieto G, Vasanthakumar A and Martino MM (2024) Tregs delivered post-myocardial infarction adopt an injury-specific phenotype promoting cardiac repair via macrophages in mice. Nat Commun 15:6480. doi: 10.1038/s41467-024-50806-y

10. Liao Z, Chen Y, Duan C, Zhu K, Huang R, Zhao H, Hintze M, Pu Q, Yuan Z, Lv L, Chen H, Lai B, Feng S, Qi X and Cai D (2021) Cardiac telocytes inhibit cardiac microvascular endothelial cell apoptosis through exosomal miRNA-21-5p-targeted cdip1 silencing to improve angiogenesis following myocardial infarction. Theranostics 11:268-291. doi: 10.7150/thno.47021

11. Hu J, Zheng Z, Li X, Li B, Lai X, Li N, Lei S ( 2021) Metformin Attenuates Hypoxia-induced Endothelial Cell Injury by Activating the AMP-Activated Protein Kinase Pathway. J Cardiovasc Pharmacol 77(6):862-874. doi: 10.1097/FJC.0000000000001028.

12. Eissner G, Hartmann I, Kesikli A, Holler E, Haffner S, Sax T, Schray C, Meiser B and Reichart B (2011) CD4+CD25+FoxP3+ regulatory T cells enhance the allogeneic activity of endothelial-specific CD8+/CD28-CTL. Int Immunol 23:485-92. doi: 10.1093/intimm/dxr041

13. Sato Y, Liu J, Lee E, Perriman R, Roncarolo MG and Bacchetta R (2021) Co-Expression of FOXP3FL and FOXP3Delta2 Isoforms Is Required for Optimal Treg-Like Cell Phenotypes and Suppressive Function. Front Immunol 12:752394. doi: 10.3389/fimmu.2021.752394

14. Zhang G, Yu K, Bao Z, Sun X and Zhang D (2021) Upregulation of FoxM1 protects against ischemia/reperfusion-induced myocardial injury. Acta Biochim Pol 68:653-658. doi: 10.18388/abp.2020_5536
